# Dynamic changes of gut microbiota between the first and second trimester for women with gestational diabetes mellitus and their correlations with BMI: a nested cohort study in China

**DOI:** 10.3389/fmicb.2024.1467414

**Published:** 2024-12-11

**Authors:** Shilin Zhong, Bingcai Yang, Yuzhen Liu, Wenkui Dai, Guanglei Li, Juan Yang, Ao Yang, Ying Wang, Min Wang, Chang Xu, Yuqing Deng

**Affiliations:** ^1^Center of Obstetrics and Gynecology, Peking University Shenzhen Hospital, Shenzhen, China; ^2^Institute of Obstetrics and Gynecology, Shenzhen PKU-HKUST Medical Center, Shenzhen, China; ^3^Shenzhen Key Laboratory on Technology for Early Diagnosis of Major Gynecologic Diseases, Shenzhen, China; ^4^CheerLand Biological Technology Co., Ltd., Shenzhen, China; ^5^Intelligent Hospital Research Academy, Peking University Shenzhen Hospital, Shenzhen, China

**Keywords:** gestational diabetes mellitus, gut microbiota, body mass index, obesity, inflammation

## Abstract

**Introduction:**

Gut microbiota (GM) has been implicated in gestational diabetes mellitus (GDM), yet longitudinal changes across trimesters remain insufficiently explored.

**Methods:**

This nested cohort study aimed to investigate GM alterations before 24 weeks of gestation and their association with GDM. Ninety-three Chinese participants provided fecal samples during the first and second trimesters. Based on oral glucose tolerance tests, 11 participants were classified as GDM, and 82 as non-diabetic (ND). Using 16S rRNA sequencing, we analyzed both cross-sectional and longitudinal differences in GM structure between those two groups.

**Results:**

In the first trimester, GDM group exhibited lower levels of *Bacteroides_H* and *Acetatifactor* compared to ND group (*p* < 0.05). In the second trimester, GDM individuals showed increased abundance of *Fusobacteriota* and *Firmicutes_D*, and genera including *Fusobacterium_A* and *Fournierella*, while *Anaerotruncus* and others decreased (*P<*0.05). Inflammation-associated genera like *Gemmiger_A_73129* and *Enterocloster* increased, while *Megamonas* decreased in overweight or obese GDM women, which was not identified in normal-weight women. The ratios of relative abundance of genera *Streptococcus*, *Enterocloster*, and *Collinsella* exceeded 1.5 in the GDM group, particularly in overweight or obese individuals. Inflammatory pathways related to African trypanosomiasis and *Staphylococcus aureus* infection were predicted to be up-regulated in overweight or obese GDM individuals but not in normal-weight GDM women.

**Discussion:**

This study suggests that GM of women with GDM undergoes significant alterations between the first and second trimesters, potentially linked to inflammation, with more pronounced changes observed in overweight or obese individuals.

## Introduction

Gestational diabetes mellitus (GDM) is defined as diabetes diagnosed in the second or third trimester of pregnancy that was not clearly overt diabetes prior to gestation (American Diabetes Association, 2020). As a prevalent obstetric complication, GDM affects over one in six pregnant women globally (He et al., 2023). This not only leads to adverse maternal outcomes such as primary cesarean delivery and preeclampsia (Catalano et al., 2012), but also impacts fetal outcomes, including hypoglycemia, macrosomia, and preterm delivery (Domanski et al., 2018). Furthermore, GDM has long-term health implications for both mothers and their offspring (Bianco and Josefson, 2019). Women with GDM face a significantly increased risk of metabolic diseases, including type 2 diabetes mellitus (T2DM) (Sheiner, 2020) and obesity. Their offspring also have a heightened risk of cardiovascular alterations (Di Bernardo et al., 2023) and diabetes (Sweeting et al., 2022; Mitanchez et al., 2015). Prenatal exposure to untreated GDM is a significant and independent risk factor for impaired glucose tolerance (IGT) in childhood (Lowe et al., 2019). Notably, GDM treatment may not substantially reduce adverse metabolic outcomes in children (Bianco and Josefson, 2019). With China’s recent fertility policy removing fertility restrictions and potentially increasing the proportion of older and multiparous women, the incidence of GDM could rise (Wang D. et al., 2024). Therefore, it is crucial to explore GDM risk factors in early pregnancy and implement preventative measures to manage its occurrence.

Increasing evidence suggests that gut microbiota (GM) plays a critical role in regulating glucose metabolism homeostasis (Palmnäs-Bédard et al., 2022). Dysbiosis in GM contributed to glucose intolerance and insulin resistance, which were linked to T2DM (Gurung et al., 2020) and GDM (Medici Dualib et al., 2021; Balleza-Alejandri et al., 2024). Mechanisms underlying those links include low-grade endotoxemia due to increased gut permeability (Ye et al., 2023), an imbalance in the production of short-chain fatty acids (Bielka et al., 2022) and branched-chain amino acids (Pedersen et al., 2016), as well as disruptions in bile acid metabolism (Cai et al., 2022). GM alterations may serve as biomarkers for diagnosing glucose metabolism abnormalities and, more importantly, as potential targets for GDM intervention (Crudele et al., 2023; Li H.-Y. et al., 2021; Hu et al., 2023). GDM may also alter the structure of breastmilk microflora, which may affect the health of offspring (Gámez-Valdez et al., 2021). Thus, examining GM characteristics and markers in women with GDM between the first trimester (FT) and second trimester (ST) could aid in diagnosing, treating, or preventing GDM.

Recently, several studies have examined the structure and diversity of GM in pregnant women with GDM (Chen F. et al., 2021; Chen T. et al., 2021; Hu et al., 2021; Zheng et al., 2020). Several differentially abundant taxa have been identified between the GDM and control groups at both genus and species levels (Chen F. et al., 2021). Changes in GM compositions during the second trimester, prior to GDM diagnosis, are linked to fasting serum metabolite levels (Chen T. et al., 2021). GM dynamics in early pregnancy differed significantly between GDM and normoglycemic women (Hu et al., 2021), suggesting potential as early biomarkers for GDM (Zheng et al., 2020). Beneficial gut microorganisms were inversely associated with GDM, while opportunistic pathogens increased GDM risk, correlating with higher OGTT glucose levels (Hu et al., 2021).

However, significant inconsistencies existed among these studies (Wang S. et al., 2024). For instance, *Streptococcus* decreased in one study (Zheng et al., 2020) but increased in another (Zhang et al., 2021), and *Lachnospiraceae* exhibited opposite trends (Hu et al., 2021; Ma et al., 2020). Such heterogeneity may stem from variations in study population characteristics, gestational age at sampling, sequencing methods, and analysis techniques, which pose challenges in cross-sectional comparisons. Importantly, only a few studies have investigated GM changes from the first to the second trimester in women with GDM (Zheng et al., 2020; Sun et al., 2023). Additionally, obesity or overweight is also significantly associated with gut microbiota (Afzaal et al., 2022), and there is a complex interaction between obesity/overweight and GDM. Therefore, a longitudinal cohort study with matching and stratification based on initial BMI is warranted to reduce the effect of confounding factors.

This nested cohort study from China aims to examine the gut microbiota characteristics in women with GDM during the first trimester (FT) and second trimester (ST). It also seeks to explore the correlation between longitudinal changes in gut microbiota and GDM, utilizing the variance of the relative abundance (VRA) and ratio of the relative abundance (RRA) indices. Additionally, stratified analysis based on the initial BMI was conducted. The findings from this study are anticipated to contribute valuable insights for the prediction, prevention, and management of GDM.

## Materials and methods

### Subjects

**Figure 1:**
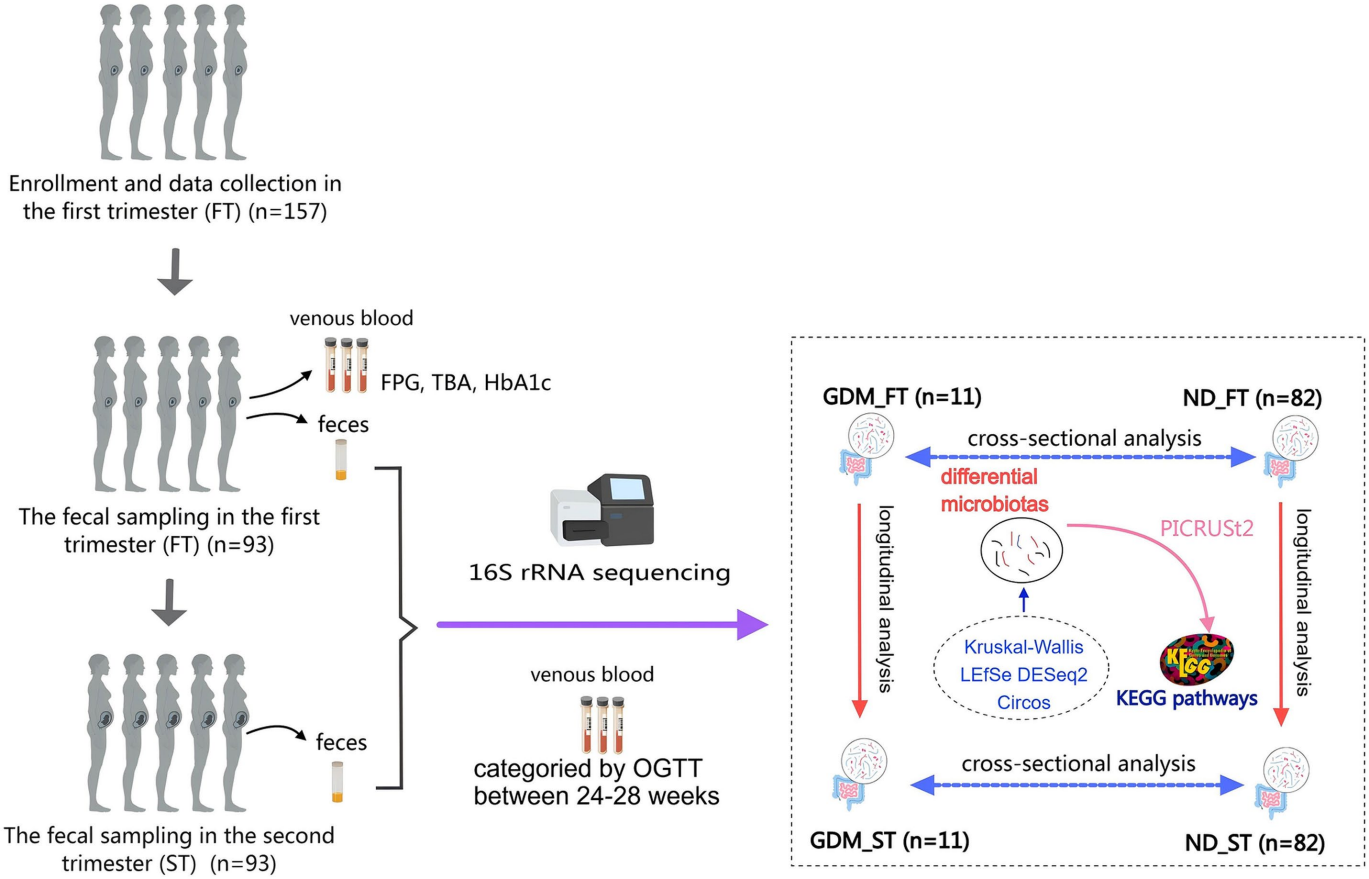
The flow chart of this study. GDM, gestational diabetes mellitus; ND, non-diabetics; HbA1c, glycated hemoglobin A1c; OGTT, oral glucose tolerance test; TBA, total bile acid; LEfSe, Linear discriminant analysis Effect Size; PICRUSt, Phylogenetic Investigation of Communities by Reconstruction of Unobserved States; KEGG, Kyoto Encyclopedia of Genes and Genomes.

This nested cohort study was conducted at Peking University Shenzhen Hospital from April 2023 to November 2023. Participants were eligible if they were aged 18–45 years, had a gestational age less than 14 weeks at enrollment, and provided informed consent. Exclusion criteria comprised pre-pregnancy diabetes, hypertension, intrahepatic cholestasis of pregnancy, chronic diarrhea, acute or chronic infectious diseases, and use of antibiotics/probiotics within the last 3 months. Participants diagnosed with GDM were assigned to the GDM group, while those without GDM were placed in the non-diabetic (ND) group (Figure 1). Based on the BMI in the first trimester, participants were further categorized into normal weight and overweight/obese subgroups within GDM and ND groups. According to a previous Chinese study, 14 cases of GDM were sufficient to demonstrate significant GM differences in the second trimester between GDM and control groups (Li et al., 2023). Given the 15% GDM incidence in China (Juan and Yang, 2020), a sample size of 93 participants were calculated. Accounting for a 15% dropout rate, a total of 110 women were recruited. This study received approval from the Ethics Committee of Peking University Shenzhen Hospital (No. 2022-007).

### GDM diagnosis and clinical information collection

A 5-mL venous blood sample was collected from all participants after a 10-h fast between 11–13 weeks and 6 days of gestation. This sample was used to measure fasting plasma glucose (FPG), total bile acids, and glycosylated hemoglobin levels. During 24–28 weeks of gestation, a 75 g oral glucose tolerance test (OGTT) was performed to determine the presence of GDM. Venous blood samples were taken at fasting (0-h), 1-h, and 2-h after consuming a 75 g glucose solution. FPG, 1-h blood glucose, and 2-h blood glucose levels during the OGTT were measured using the hexokinase method with a Glucose assay kit on a Beckman AU5800 analyzer (Agencourt, Beckman Coulter, Brea, CA, United States). GDM was diagnosed according to the criteria of the International Association of Diabetes and Pregnancy Study Group (IADPSG) (He et al., 2022), where OGTT 0-h blood glucose ≥5.1 mmol/L, 1-h blood glucose ≥10.0 mmol/L, or 2-h blood glucose ≥8.5 mmol/L indicated GDM. HbA1c was measured using the high-performance liquid chromatography (HPLC) method on a Glycosylated hemoglobin analyzer D-100 (Bio-Rad Laboratories, Berkeley, CA, United States) with an HbA1c Kit. Total bile acids (TBA) were examined using the Total Bile Acid Assay Kit (Leadman bio, Beijing, China) with the enzymatic cycling method on a Beckman AU5800 analyzer.

Body weight was recorded in the first trimester (11–14 weeks) and second trimester (21–24 weeks), with BMI calculated as weight (kg) divided by height (m^2^). BMI in the first trimester between 18.5 and 24 kg/m^2^ was considered normal weight, while BMI ≥ 24 kg/m^2^ indicated overweight or obesity. Data on maternal age, ethnicity, parity, occupation, and education were also collected and compared between the GDM and ND groups.

### Specimen sampling

Fecal samples were collected using a disposable sampling kit (Miraclean Tech Co., Ltd., Shenzhen, China) during the first trimester (11 weeks to 13 weeks and 6 days) and the second trimester (21 week to 23 weeks and 6 days). Over 20 grams of feces were collected under aseptic conditions and immediately placed into cryotubes containing preservation solution. Samples were stored at −80°C within 24 h and transported to the laboratory (Wekemo Tech Group Co., Ltd., Shenzhen, China) for subsequent testing. During transport, the samples were kept on dry ice.

### 16S rRNA gene sequencing

Total fecal genomic DNA was extracted from approximately 250 mg-500 mg of feces using Magnetic Stool DNA Kit (Cat# DP712, TIANGEN Biotech Co. Ltd., Beijing, China), following the manufacturer’s instructions. After concentrating and purifying the DNA, primers 515F (5’-GTGCCAGCMGCCGCGGTAA-3′) and 907R (5’-CCGTCAATTCCTTTGAGTTT-3′) were used to amplify the V4 to V5 regions of 16S rRNA genes via PCR. PCR products were purified using a Universal DNA Kit (Cat# DP214, TianGen, China). Sequencing libraries were generated using the NEBNext® Ultra DNA Library Prep Kit (Cat# E7370L, Illumina, San Diego, CA, United States), following the manufacturer’s guidelines. Library quality was assessed using the Agilent 5400 system (Agilent Technologies Co. Ltd., United States). Sequencing was performed on an Illumina platform, producing 250 bp paired-end reads.

### Bioinformation analysis

The analysis followed the QIIME2 tutorial.^1^ Raw FASTQ files were imported into a QIIME2-compatible format using the QIIME tools import program. Sequences from each sample were demultiplexed and filtered for high quality, trimmed, and de-noised. Chimeric sequences were identified and removed using the QIIME2 dada2 plugin to obtain the amplicon sequence variant (ASV) feature table. ASV sequences were aligned to the pre-trained Greengenes2 database^2^ (McDonald et al., 2024) using the QIIME2 feature-classifier plugin to generate a taxonomy table. Contaminating mitochondrial and chloroplast sequences were filtered out. Relative abundance (RA) was used to reflect the proportion of a certain genus or phylum in the total microbiota, with bacterial flora having RA > 0.1% defined as dominant flora. Kruskal-Wallis, Linear discriminant analysis Effect Size (LEfSe), DESeq2, and Circos analysis were applied to identify bacteria with different RA among groups (Figure 1). Diversity metrics were calculated using the core-diversity plugin within QIIME2. Alpha diversity was assessed using the Chao1 and Shannon indices, while beta diversity was investigated using nonmetric multidimensional scaling (NMDS) and principal coordinate analysis (PCoA). Phylogenetic Investigation of Communities by Reconstruction of Unobserved States (PICRUSt) (Langille et al., 2013) predicted potential functional Kyoto Encyclopedia of Genes and Genomes (KEGG) pathways associated with changes in gut microbiota composition in the GDM group (Figure 1). Samples were divided into first-trimester GDM (GDM_FT), first-trimester ND (ND_FT), second-trimester GDM (GDM_ST), and second-trimester ND (ND_ST) groups for microbiota abundance comparison.

In this study, variance of the relative abundance (VRA) and ratio of the relative abundance (RRA) were used to analyze microbiota differences between the first and second trimesters in the GDM and ND groups. VRA was defined as the difference in RA between the second and first trimesters for a given bacterial flora, with the average VRA compared between the GDM and ND groups. RRA was calculated as the ratio of the mean RA in the second trimester to the mean RA in the first trimester in the GDM group, divided by the corresponding ratio in the ND group, using the formula: RRA = (RA^GDM_ST^ / RA^GDM_FT^) / (RA^ND_ST^ / RA^ND_FT^). RRA reflects the change in VR of a given flora between trimesters, adjusted for background changes. An RRA > 1.5 or < 0.8 was considered statistically significant.

### Statistical analysis

Statistical analysis was performed using SPSS 26.0 software (SPSS Inc., Chicago, IL, United States). Categorical variables were expressed as frequencies and compared using the chi-square test. Quantitative data following a normal distribution were presented as mean ± standard deviation and compared using the *t*-test. For non-normally distributed data, the median (Q1, Q3) was provided, and comparisons were made using the Mann–Whitney *U* test or Kruskal-Wallis test. For relative abundance comparisons, *p*-values or Q-values (false discovery rate adjusted) < 0.05 were considered significant. In the LEfSe analysis, microbiotas with linear discriminant analysis (LDA) scores >2 were identified as potential biomarkers. Statistical significance was defined as *p <* 0.05.

## Results

### Clinical data

Of the 157 pregnant women who met the inclusion criteria, 93 successfully provided fecal samples in both the first and second trimesters and were included in the study (Figure 1). The participants’ ages ranged from 19 to 42 years, with an average age of 31.7 ± 3.4 years. The gestational age at the time of the first sample collection ranged from 11 weeks and 3 days to 13 weeks and 6 days, while for the second sample, it ranged from 21 weeks to 23 weeks and 5 days. In the second trimester, 11 women (11.83%) were diagnosed with GDM. The GDM group exhibited significantly higher BMIs and OGTT blood glucose levels compared to ND group (*p* < 0.05). However, no significant differences were observed between the groups in terms of maternal age, ethnicity, parity, occupation, educational status, gestational weeks at either sampling time point, fasting plasma glucose, total bile acid, or glycosylated hemoglobin levels (Table 1).

**Table 1:** Comparison of clinical characteristics of the participants between GDM group and ND group.

	GDM group (*n* = 11)	ND group (*n* = 82)	*t* or *χ*^2^	*p*
Age (years)	32.63 ± 3.71	31.56 ± 3.40	0.976	0.332
Ethnicity, *n* (%)				
Ethnic Han of China	11 (100.00)	82 (100.00)	–	–
Ethnic Minority of China	0 (0.00)	0 (0.00)		
Parity, n (%)				
Primipara	6 (54.55)	60 (73.17)	–	0.287^*^
Multipara	5 (45.45)	22 (26.83)		
Occupation, *n* (%)				
Employed	10 (90.91)	80 (97.56)	–	0.318^*^
Unemployed	1 (9.09)	2 (2.44)		
Educational status, *n* (%)				
Secondary	1 (9.09)	3 (3.80)	–	0.401^*^
Tertiary	10 (90.91)	79 (96.20)		
Gestational weeks of the first sampling (weeks)	12.73 ± 0.56	12.53 ± 0.43	1.355	0.179
Gestational weeks of the second sampling (weeks)	22.05 ± 0.40	22.20 ± 0.82	0.612	0.542
HbA1c of the first sampling (%)	5.46 ± 0.64	5.06 ± 0.27	1.987	0.073
TBA of the first sampling (μmol/L)	1.427 ± 0.59	1.703 ± 1.20	0.745	0.458
Fasting plasma glucose of the first trimester (mmol/L)	4.61 ± 0.50	4.40 ± 0.37	1.670	0.099
BMI of the first sampling (kg/m^2^)	25.16 ± 5.51	20.95 ± 2.85	2.493	0.030
BMI of the second sampling (kg/m^2^)	26.51 ± 5.22	22.89 ± 3.21	3.238	0.002
Blood glucose of OGTT-0 h (mmol/L)	4.69 ± 0.64	4.22 ± 0.28	2.383	0.037
Blood glucose of OGTT-1 h (mmol/L)	10.09 ± 1.19	7.08 ± 1.24	7.573	<0.001
Blood glucose of OGTT-2 h (mmol/L)	8.97 ± 1.31	6.49 ± 0.94	7.781	<0.001

GDM, gestational diabetes mellitus; ND, non-diabetics; HbA1c, glycated hemoglobin A1c; OGTT, oral glucose tolerance test; TBA, total bile acid; BMI, body mass index; ^*^ by Fisher’s exact test.

### 16S rRNA sequencing results

Sequencing the 16S rRNA genes from 186 fecal samples produced a total of 21,280,018 high-quality reads, averaging 114,408.7 clean reads per sample. Using a 100% similarity threshold, clean reads were clustered via sklearn species classification, resulting in 80,829 amplicon sequence variants (ASVs). These ASVs were subsequently matched to the Greengenes2 database for taxonomic annotations. At a confidence coefficient of 0.7, all the ASVs were identified, with 60,908 classified at the genus level.

### Dominant microbiotas in the gut during pregnancy

At the phylum level, the dominant microbiotas (with abundance over 1%) were *Bacteroidota* (43.59%), *Firmicutes_A* (36.28%), *Proteobacteria* (7.19%), *Firmicutes_C* (5.37%), *Actinobacteriota* (2.97%), and *Firmicutes_D* (2.83%), collectively accounting for approximately 98.24% of the total microbiota. In the first trimester, *Bacteroidota* (47.75%) and *Firmicutes_A* (34.53%) were the most prevalent phyla. In the second trimester, *Bacteroidota* (39.43%) and *Firmicutes_A* (38.03%) remained dominant (Supplementary Table S1).

At the genus level, the dominant genera (with relative abundance over 1%) were *Phocaeicola_A_858004*, *Prevotella*, *Faecalibacterium*, *Bacteroides_H*, *Gemmiger_A_73129*, *Escherichia_710834*, *Lachnospira*, *Bifidobacterium_388775*, *Blautia_A_141781*, *Acinetobacter*, *Megamonas*, *Collinsella*, *Agathobacter_164117*, *Phascolarctobacterium_A*, *Parabacteroides_B_862066*, *Dialister*, *Anaerostipes*, *Fusicatenibacter*, *Streptococcus*, *Enterocloster*, *Acetatifactor*, *Roseburia*, and *Klebsiella_724518*, which together constituted 74% of the total abundance. In the first trimester, the most abundant genera (>5%) were *Phocaeicola_A_858004*, *Prevotella*, *Faecalibacterium*, and *Bacteroides_H*. In the second trimester, *Gemmiger_A_73129* joined this group (>5%) (Supplementary Table S2).

### Differential GM of GDM women in the first trimester varied based on BMI status

**Figure 2:**
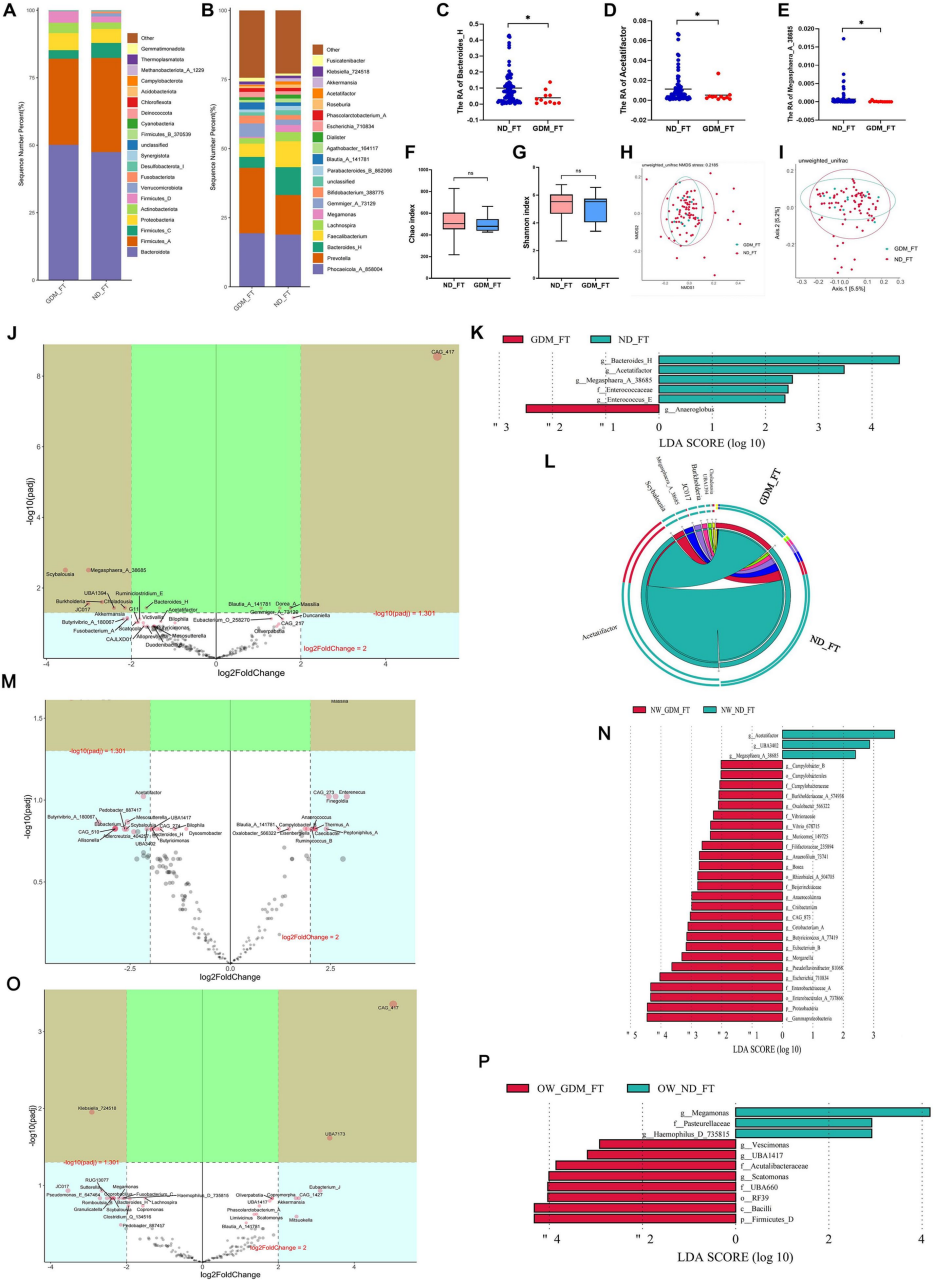
Comparison of the structure and diversity of gut microbiota between the GDM and ND groups in the first trimester. The dominant phyla **(A)** and genera **(B)** differed between ND and GDM groups in FT. *Bacteroides_H* **(C)**, *Acetatifactor* **(D)**, and *Megasphaera_A_38685* **(E)** were significantly lower in the GDM group (*p <* 0.05). No significant differences were found in the Chao **(F)** or Shannon indices **(G)**. NMDS **(H)** and PCoA **(I)** showed no distinct microbiota separation. The volcano plot **(J)** highlighted six genera that increased and one that decreased significantly in the GDM group. LEfSe analysis **(K)** identified one biomarker for GDM and five for ND in FT. The Circos plot **(L)** showed a closer relationship between *Acetatifactor*, *Scybalousia*, *Megasphaera_A_38685*, and *Burkholderia* with ND. Significant differential genus related to GDM were identified in participants with normal weight **(M)**, and those overweight or obese **(O)**. LEfSe analysis also pinpointed biomarkers for both groups among participants who were normal weight **(N)** and those overweight or obese **(P)**. * *p <* 0.05.

In the first trimester, the GDM group showed slight, non-significant variations in the dominant phyla compared to the ND group (Figure 2A). While *Prevotella* increased in the GDM group, this increase was not statistically significant (Figure 2B). However, *Bacteroides_H* (*p* = 0.020) (Figure 2C), *Acetatifactor* (*p* = 0.049) (Figure 2D), and *Megasphaera_A_38685* (*p* = 0.018) (Figure 2E) significantly decreased in the GDM group. Alpha diversity (Figures 2F,G) and beta diversity (Figures 2H,I) showed no significant differences between the groups. The volcano plot indicated a significant increase in CAG_417 and a decrease in *Scybalousia*, *Megasphaera_A_38685*, *UBA1394*, *Burkholderia*, *Choladousia*, and *JC017* in the GDM group (Figure 2J). *Anaeroglobus* was identified as a GDM marker, while *Bacteroides_H*, *Acetatifactor*, *Megasphaera_A_38685*, *Enterococcaceae*, and *Enterococcus_E* were markers for the ND group (Figure 2K). The Circos plot suggested a weaker association of *Acetatifactor*, *Scybalousia*, *Megasphaera_A_38685*, and *Burkholderia* with GDM (Figure 2L).

In normal-weight women, the GDM group showed a significant increase in *Escherichia_710834*, *Blautia_A_141780*, *Massilia*, *Thermus_A*, *Campylobacter_B*, *Oxalobacter_566322*, and *Slackia_A*, while *Megasphaera_A_38685*, *UBA3402*, and *Acetatifactor* decreased (*p <* 0.05) (Supplementary Table S3). The volcano plot highlighted a significant increase in *Massilia* and a decrease in *Megasphaera_A_38685* (Figure 2M). Linear discriminant analysis (LDA) identified 26 marker bacterial communities in the GDM group and 3 in the ND group (Figure 2N).

In overweight or obese GDM women, the abundance of *Vescimonas*, *UBA1417*, *CAG_510*, *Gordonibacter*, *Brevundimonas*, *Eubacterium_O_258270*, and *Scatomonas* significantly increased, while *Haemophilus_D_735815* decreased (*p <* 0.05) (Supplementary Table S4). *CAG_417* and *UBA7173* were notably higher, and *Klebsiella_724518* was lower in GDM patients, as shown by volcano plot (Figure 2O). LDA identified 8 marker bacterial communities in the GDM group and 3 in the ND group (Figure 2P). No significant difference in microbiota diversity was observed between the GDM and ND groups, regardless of BMI, in the first trimester (Supplementary Figures S1–S4).

### Differential GM of GDM women in the second trimester varied based on BMI status

**Figure 3:**
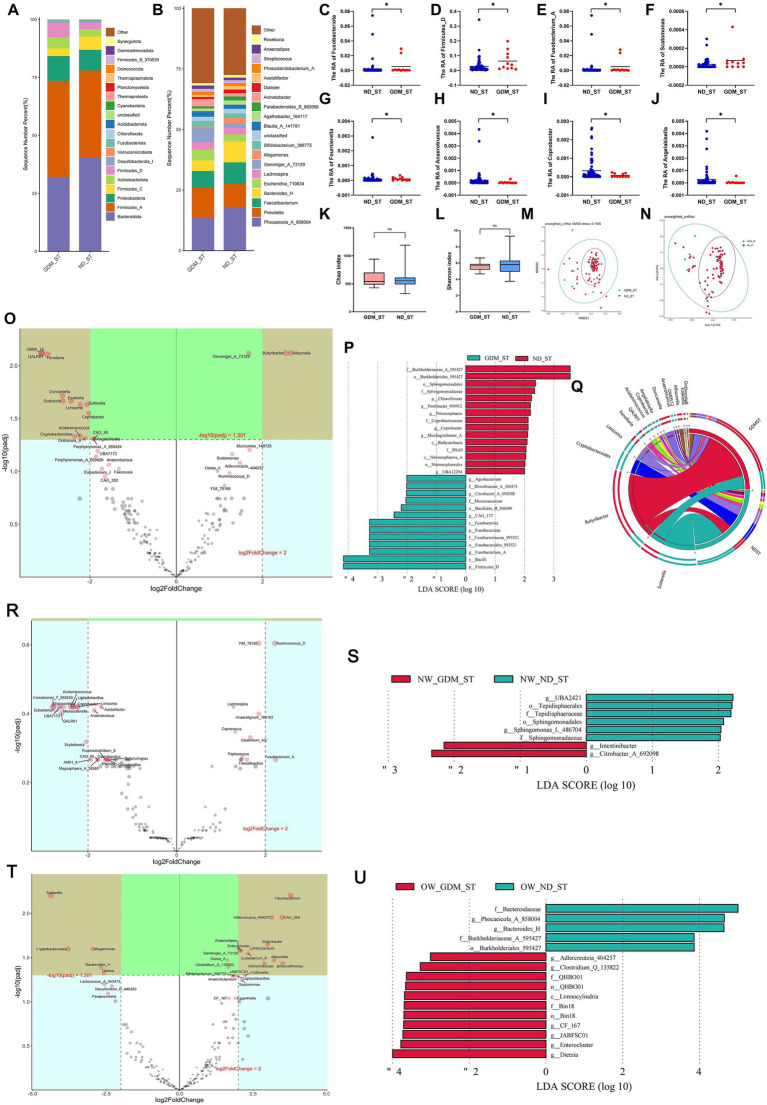
Comparison of the structure and diversity of gut microbiota between the GDM and ND groups in the second trimester. The proportion of dominant phyla **(A)** and genera **(B)** varied between the ND and GDM groups in the second trimester (ST). At the phylum level, *Fusobacteriota* **(C)** and *Firmicutes_D* **(D)** significantly decreased in GDM group. At the genus level, *Fusobacterium_A* **(E)**, *Scatomonas* **(F)**, and *Fournierella* **(G)** significantly increased, whereas *Anaerotruncus* **(H)**, *Coprobacter* **(I)**, and *Angelakisella* **(J)** significantly decreased in GDM group. No significant differences were noted in the Chao **(K)** or Shannon indices **(L)**. NMDS **(M)** and PCoA **(N)** showed no microbiota separation between groups. The volcano plot **(O)** showed two genera increased and twelve decreased in GDM. LEfSe analysis **(P)** identified thirteen GDM biomarkers and fifteen ND biomarkers. The Circos plot **(Q)** highlighted close relationships between *Butyribacter*, *Duncaniella*, and *Allisonella* with GDM. The volcano plot showed no significant differential genera related to GDM in normal-weight participants **(R)**, but overweight or obese women had increases in *Enterocloster*, *Gemmiger_A_73129*, and nine other genera, and decreases in *Megamonas*, *Bacteroides_H*, and three others **(T)**. LEfSe analysis identified biomarker for the GDM group and ND group in the participants with normal weight **(S)**, overweight or obese **(U)**. *p < 0.05.

In the second trimester, the composition of dominant phyla (Figure 3A) and genera (Figure 3B) differed between the GDM and ND groups. At the phylum level, *Fusobacteriota* (Figure 3C) and *Firmicutes_D* (Figure 3D) were significantly higher in the GDM group (*p <* 0.05). At the genus level, *Fusobacterium_A* (Figure 3E), *Scatomonas* (Figure 3F), and *Fournierella* (Figure 3G) significantly increased, while *Anaerotruncus* (Figure 3H), *Coprobacter* (Figure 3I), and *Angelakisella* (Figure 3J) significantly decreased in the GDM group (*p <* 0.05). However, alpha diversity (Figures 3K,L) and beta diversity (Figures 3M,N) did not significantly differ between the GDM and ND groups. The Volcano plot (Figure 3O) showed that in the second trimester, *Butyribacter* and *Allisonella* were significantly increased, while *OM05_12*, *QALR01*, *Fenollaria*, *Duncaniella*, *Scatocola*, *Ezakiella*, *Limisoma*, *Sutterella*, *Coprobacter*, *Acidaminococcus*, *Cryptobacteroides*, and *Onthocola_B* were significantly decreased in the GDM group. LEfSe analysis identified 13 microbiotas as biomarkers for the GDM group and 15 for the ND group (Figure 3P). The Circos plot suggested a close association between *Butyribacter*, *Duncaniella*, and *Allisonella* with the GDM group (Figure 3Q).

In normal-weight women, *Christensenella*, *Coprenecus*, and *Intestinibacter* were significantly more abundant in the GDM group during the second trimester (*p <* 0.05) (Supplementary Table S5). However, no significant difference was found between the GDM and ND groups in the volcano plot (Figure 3R). LDA identified 2 marker bacterial communities in the GDM group and 6 in the ND group (Figure 3S).

For overweight or obese participants, *Enterocloster*, *Clostridium_Q_135822*, and *Adlercreutzia_404257* increased, whereas *Dielma*, *Phocaeicola_A_858004*, and *Bacteroides_H* decreased in the GDM group (Supplementary Table S6). The volcano plot indicated significant increases in *Flavobacterium*, *Adlercreutzia_404257*, *CAG_269*, *Butyribacter*, *Asticcacaulis*, *Enterocloster*, *Gemmiger_A_73129*, *Eubacterium_R*, *Allisonella*, *Brevundimonas*, and *Achromobacter*, while *Sutterella*, *Cryptobacteroides*, *Megamonas*, *Bacteroides_H* and *Dielma* significantly decreased (Figure 3T). LDA identified 11 marker communities in the GDM group and 5 in the ND group (Figure 3U). No significant difference in GM alpha or beta diversity was observed between the GDM and ND groups in the second trimester, regardless of initial BMI (Supplementary Figures S5–S8).

### Some inflammation-related microbes showed significant increase from the first to the second trimester in overweight/obese GDM women

Matched comparative analyses were used to investigate longitudinal changes in the microbiota from the first trimester (FT) to the second trimester (ST). In the GDM group, the relative abundance of 14 genera during the second trimester was markedly higher than in the first trimester; however, only 8 of these genera showed a significant increase in the ND group. Specifically, the abundance of *Streptococcus* rose from 0.288 to 1.308% in the GDM group, compared to an increase from 0.521 to 1.166% in the ND group (Supplementary Table S7).

**Figure 4:**
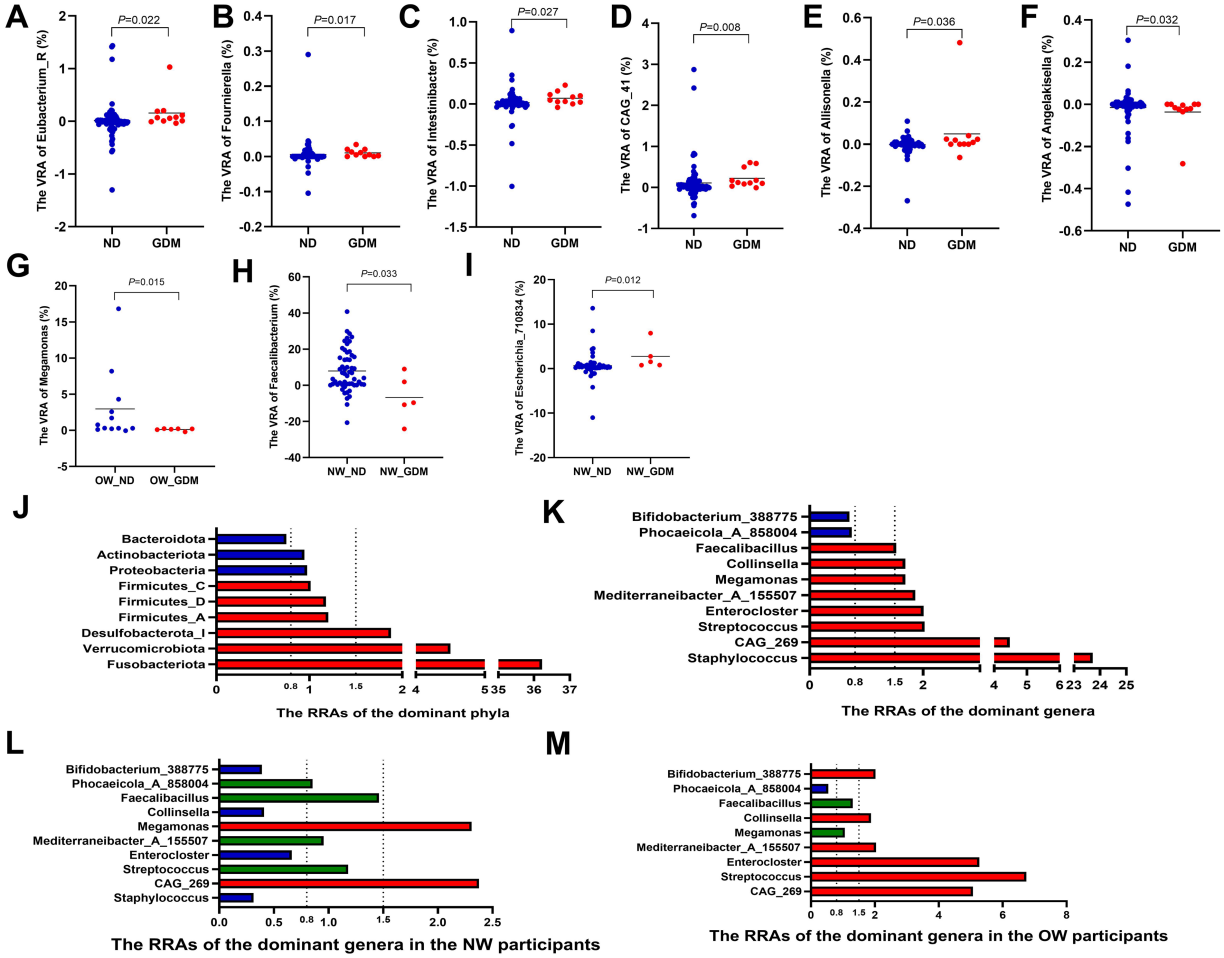
Variations in the relative abundance of dominant phyla and genera between the GDM and ND groups. The VRAs of *Eubacterium_R* **(A)**, *Allisonella* **(B)**, *Fournierella* **(C)**, *Intestinibacter* **(D)** and *CAG_41* **(E)** increased, while *Angelakisella* **(F)** decreased in GDM group. Overweight or obese GDM women showed increased VRA of *Megamonas* **(G)**, whereas normal-weight GDM women had higher VRA of *Faecalibacterium* **(H)** and lower VRA of *Escherichia_710834* **(I)**. Phyla *Fusobacteriota*, *Verrucomicrobiota*, and *Desulfobacterota_I* had RRAs over 1.5, while *Bacteroidota* was below 0.8 **(J)**. Genera *Staphylococcus*, *Streptococcus*, *Enterocloster*, *Collinsella*, and *Faecalibacillus* exceeded 1.5, with *Phocaeicola_A_858004* and *Bifidobacterium_388775* below 0.8 **(K)**. In normal-weight women, *Staphylococcus*, *Enterocloster*, and *Collinsella* had RRAs less than 0.8, with *Streptococcus*, *Mediterraneibacter_A_155507*, and *Faecalibacillus* between 0.8 and 1.5, and only *CAG_269* and *Megamonas* above 1.5 **(L)**. In overweight or obese women, RRAs of *CAG_269*, *Streptococcus*, *Enterocloster*, *Mediterraneibacter_A_155507*, and *Collinsella* exceeded 1.5 **(M)**. RA, relative abundance; VRA, variance of the relative abundance from the first to the second trimester; RRA, ratio of the relative abundance. * *p* < 0.05.

At the genus level, compared with the ND group, the variances of relative abundance (VRAs) of *Eubacterium_R* (Figure 4A), *Allisonella* (Figure 4B), *Fournierell*a (Figure 4C), *Intestinibacter* (Figure 4D), and *CAG_41* (Figure 4E) significantly increased in the GDM group (*p <* 0.05). Conversely, *Angelakisella* (Figure 4F) exhibited a significant decrease in VRA (*p <* 0.05) (Supplementary Table S8). In overweight or obese participants, the VRA of *Megamonas* in the GDM group was significantly lower than in the ND group (Figure 4G), whereas this difference was not observed in women of normal weight. Among normal-weight women, the VRA of *Faecalibacterium* was higher (Figure 4H), while the VRA of *Escherichia_710834* was lower (Figure 4I), compared to the ND group.

The relative abundance ratios (RRAs) of *Fusobacteriota*, *Verrucomicrobiota*, and *Desulfobacterota_I* in the GDM group all exceeded 1.5 (36.22, 4.50, and 1.88, respectively) (Figure 4I), indicating a higher magnitude of increase from the first to the second trimester in the GDM group compared to the ND group. In contrast, the RRA of *Bacteroidota* was only 0.75, indicating a lesser change in relative abundance in the GDM group (Figure 4J; Supplementary Table S9). At the genus level, the RRAs of *Staphylococcus*, CAG_269, *Streptococcus*, *Enterocloster*, *Mediterraneibacter_A_155507*, *Megamonas*, *Collinsella*, and *Faecalibacillus* were all greater than 1.5 (23.72, 4.48, 2.03, 2.01, 1.86, 1.69, 1.69, and 1.53, respectively), while the RRAs of *Phocaeicola_A_858004* and *Bifidobacterium_388775* were both less than 0.8 (0.74 and 0.70, respectively) (Figure 4K; Supplementary Table S10).

In normal-weight women, the RRAs of *Staphylococcus*, *Enterocloster* and *Collinsella* were all less than 0.8. The RRAs of *Streptococcus*, *Mediterraneibacter_A_155507* and *Faecalibacillus* were between 0.8 and 1.5, while only *CAG_269* and *Megamonas* maintained RRAs greater than 1.5 (Figure 4L). However, in overweight or obese women, the RRAs of *CAG_269*, *Streptococcus*, *Enterocloster*, *Mediterraneibacter_A_155507*, and *Collinsella* were all larger than 1.5 (Figure 4M).

### Variations in the relative abundance of microbiota from the first to second trimesters linked to blood glucose and HbA1c levels

**Figure 5:**
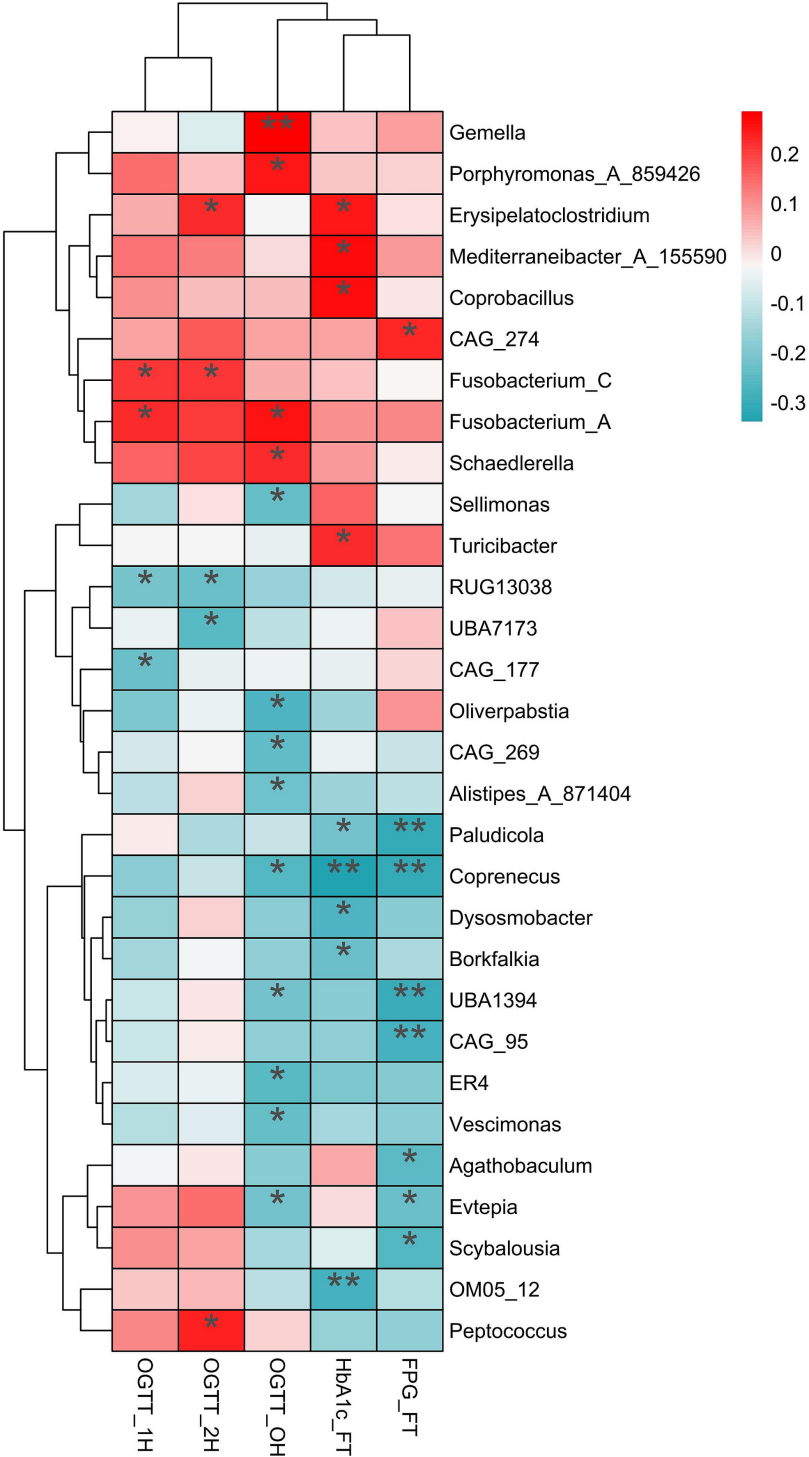
The relationship of variances of relative abundance with the blood glucose and HblAc. The significantly positive relationship (red) and negative relationship (dark turquoise) were label by asterisk. OGTT, oral glucose tolerance test; HblAc, glycated hemoglobin A1c; FPG, fasting plasma glucose; * *p* < 0.05, **p < 0.01.

Spearman analysis revealed positive correlations between the variances of relative abundance (VRAs) of certain microbiotas and blood glucose levels during OGTT. Specifically, *Fusobacterium_A* (*r =* 0.258, *p =* 0.015), *Gemella* (*r =* 0.285, *p =* 0.007), *Schaedlerella* (*r =* 0.225, *p =* 0.035) and *Porphyromonas_A_859426* (*r =* 0.251, *p =* 0.018) showed positive relationships with OGTT-0 h blood glucose levels. Similarly, *Fusobacterium_A* (*r =* 0.223, *p =* 0.037) and *Fusobacterium_C* (*r =* 0.211, *p =* 0.048) were positively correlated with OGTT-1 h blood glucose levels, while *Erysipelatoclostridium* (*r =* 0.224, *p =* 0.036), *Fusobacterium_C* (*r =* 0.216, *p =* 0.043), and *Peptococcus* (*r =* 0.240, *p =* 0.025) were positively correlated with OGTT-2 h blood glucose levels (Figure 5).

Additionally, the VRAs of *Erysipelatoclostridium* (*r =* 0.251, *p =* 0.020), *Mediterraneibacter_A_155590* (*r =* 0.271, *p =* 0.012), *Coprobacillus* (*r =* 0.261, *p =* 0.016), and *Turicibacter* (*r =* 0.227, *p =* 0.037) positively correlated with HbA1c levels in the first trimester (*p <* 0.05). Conversely, the VRAs of *Sellimonas* (*r =* −0.235, *p =* 0.028), *Oliverpabstia* (*r =* −0.265, *p =* 0.013), *CAG_269* (*r =* −0.243, *p =* 0.023), *Alistipes_A_871404* (*r =* −0.225, *p =* 0.035), *Coprenecus* (*r =* −0.259, *p =* 0.015), and *Vescimonas* (*r =* −0.232, *p =* 0.029) were negatively related to OGTT-0 h blood glucose (Figure 5).

### The inflammatory pathways up-regulated in overweight or obese women with GDM

**Figure 6:**
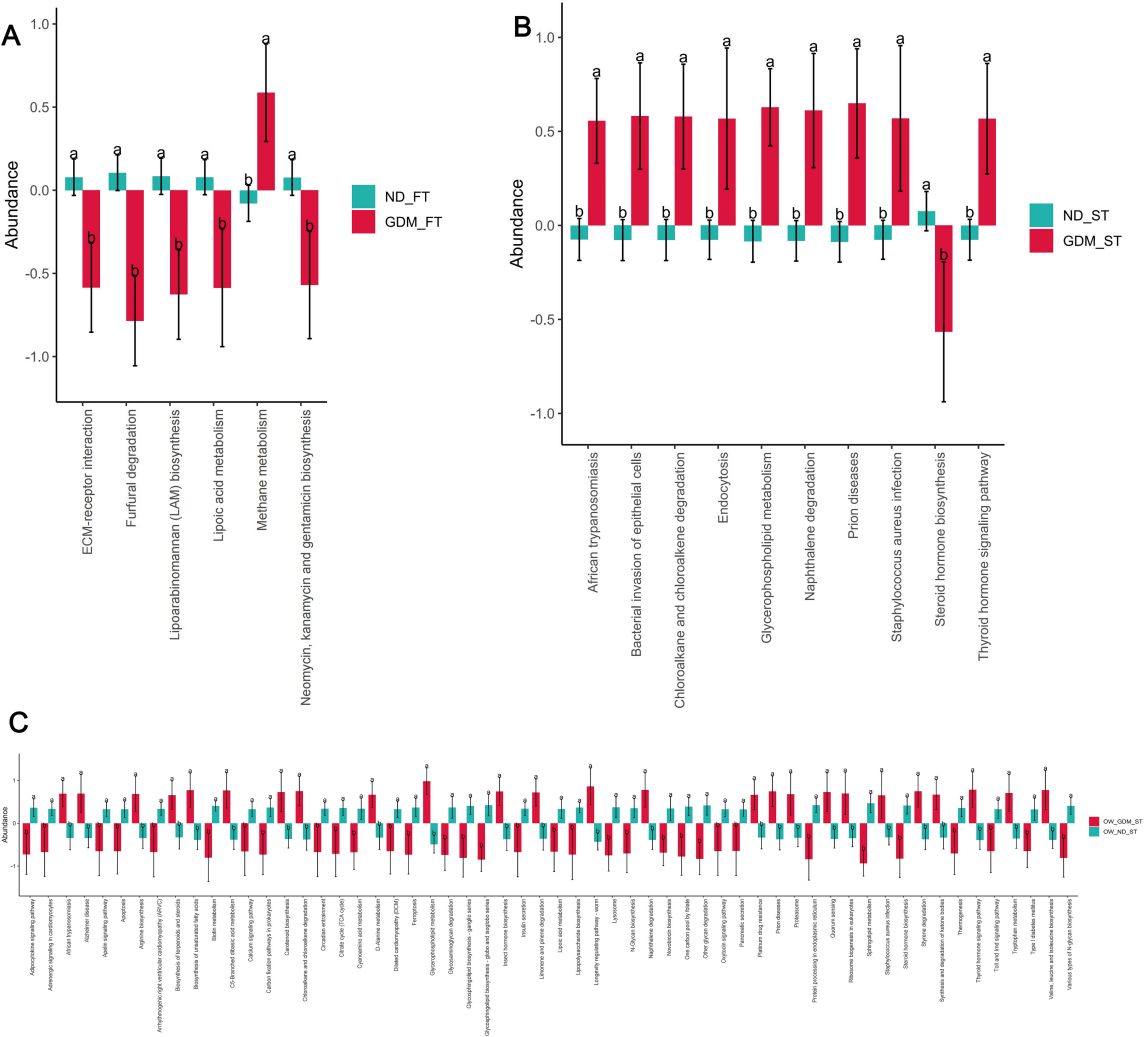
The KEGG pathways predicted by PICRUSt2 differed between the GDM and ND groups. The up-regulated KEGG pathways (red) and down-regulated pathways (dark turquoise) were predicted in the total participants of the first trimester **(A)**, second trimester **(B)** and the overweight or obese participants in the second trimester **(C)**. ND, non-diabetic; GDM, gestational diabetes mellitus; FT, first trimester; ST, second trimester; ECM, extracellular matrix. * *p* < 0.05, **p < 0.01.

PICRUSt2 software analysis predicted differences in KEGG pathways between the groups. In the first trimester, one pathway [Methane metabolism (*p =* 0.030)] was significantly up-regulated, while five KEGG pathways [ECM-receptor interaction (*p =* 0.022), Furfural degradation (*p =* 0.002), Lipoarabinomannan (LAM) biosynthesis (*p =* 0.003), Lipoic acid metabolism (*p =* 0.038), Neomycin, kanamycin and gentamicin biosynthesis (*p =* 0.026)] were significantly down-regulated in the GDM group compared to the ND group (Figure 6A).

In the second trimester, however, nine pathways, including African trypanosomiasis (*p =* 0.018), Bacterial invasion of epithelial cells (*p =* 0.016), Chloroalkane and chloroalkene degradation (*p =* 0.034), Endocytosis (*p =* 0.038), Glycerophospholipid metabolism (*p =* 0.006), Naphthalene degradation (*p =* 0.008), Prion diseases (*p =* 0.013), *Staphylococcus aureus* infection (*p =* 0.042), and Thyroid hormone signaling pathway (*p =* 0.016), were up-regulated in the GDM group. Concurrently, one pathway—Steroid hormone biosynthesis (*p =* 0.022)—was down-regulated in the GDM group. Notably, four of the nine up-regulated pathways were related to inflammation: African trypanosomiasis, bacterial invasion of epithelial cells, prion diseases, and *Staphylococcus aureus* infection (Figure 6B).

PICRUSt2 did not predict any pathways with significant between-group differences among individuals with normal weight during the first trimester (Supplementary Table S11). However, among overweight or obese individuals in the first trimester, PICRUSt2 predicted 25 significantly up-regulated KEGG pathways and 33 significantly down-regulated pathways in the GDM group (Figure 6C). Among the significantly up-regulated KEGG pathways, two were inflammation-related: African trypanosomiasis (*p =* 0.012) and *Staphylococcus aureus* infection (*p =* 0.046) (Figure 6C).

## Discussion

This nested cohort study delved into the cross-sectional characteristics of gut microbiota in pregnant women with gestational diabetes mellitus (GDM) and examined the longitudinal changes from the first to the second trimester. Certain gut genera identified early in pregnancy distinguished GDM from non-diabetic women, with more distinct microbiota profiles emerging in the second trimester. Critically, the longitudinal changes in gut microbiota, rather than merely cross-sectional characteristics, differed significantly based on GDM diagnosis. Compared to non-diabetic women, those with GDM showed significant microbiota shifts potentially linked to inflammation, particularly in overweight or obese individuals. These findings may provide insights into the connection between GDM and gut microbiota.

Previous studies have extensively examined the characteristics of intestinal flora in women with GDM across different trimesters. During the first trimester, the families *Ruminococcaceae UCG* (Ma et al., 2020) and genera *Bifidobacterium* (Chen F. et al., 2021; Hu et al., 2021; Zhang et al., 2021), and *Prevotella* (Pinto et al., 2023; Vavreckova et al., 2022) were all reduced in GDM women, whereas *Ruminococcus gnavus* (Li et al., 2023) increased in GDM. However, the relationship between some microbiotas and GDM varied across studies. In Zheng et al.’s (2020) study, *Streptococcus* was reduced in GDM, while in Zhang et al.’s (2021) study, it increased. *Lachnospiraceae* also exhibited opposite trends in different studies (Hu et al., 2021; Ma et al., 2020). In the first trimester, our study observed decreased levels of *Bacteroides_H* and *Acetatifactor* in the GDM group, identified as biomarkers for the non-diabetic group. These findings contrast with one previous study (Dualib et al., 2022) but align with another (Li et al., 2023), emphasizing the heterogeneity in gut microbiota profiles across different populations and methodologies. The decrease in *Bacteroides_H* and *Acetatifactor*, both known producers of lithocholic acid (Pathak et al., 2018), suggests abnormalities in bile acid-metabolizing bacteria that may influence glucose metabolism. *Bacteroides* is suggested to be a harmful bacterium associated with obesity (Ponzo et al., 2019) and *Acetatifactor* was increased in animal models with a high-fat diet (Zhu et al., 2023). Given that the Greengenes2 annotation database used in this study was recently updated (McDonald et al., 2024), *Bacteroides_H* annotated by this new database has not been previously reported as related to GDM. Further investigation is warranted to elucidate the correlation between these genera and GDM in early pregnancy.

Moving into the second trimester, previous studies have consistently showed that phyla *Bifidobacterium* (Chen F. et al., 2021) and *Actinobacteria* (Su et al., 2021; Chen T. et al., 2021), and genera *Lachnospiraceae* (Chen T. et al., 2021; Wang et al., 2020), *Eubacterium* (Chen F. et al., 2021; Su et al., 2021), *Prevotella* (Chen F. et al., 2021; Ye et al., 2023), *Collinsella* (Su et al., 2021; Tanaka et al., 2022), and *Faecalibacterium* (Ye et al., 2019, 2023) decreased in GDM patients, while *Blautia* (Chen F. et al., 2021; Ye et al., 2019), *Parabacteroides* (Su et al., 2021; Kuang et al., 2017), and *Megamonas* (Ye et al., 2023; Kuang et al., 2017) increased. However, phylum *Bacteroidetes*, *Verrucomicrobia*, and genera *Bacteroides*, *Subdoligranulum*, *Eggerthella*, and *Clostridium* manifested inconsistent results across studies (Wang S. et al., 2024). In our study, phyla *Fusobacteriota* and *Firmicutes_D*, as well as genera *Fusobacterium_A*, *Scatomonas*, and *Fournierella*, all increased in the GDM group during the second trimester. LEfSe analysis indicated that *Firmicutes_D* and *Fusobacteria* were markers of GDM. Spearman analysis revealed a positive correlation between *Fusobacterium_A* and OGTT-0 h and 1 h glucose levels. The changes of *Firmicutes* in our study align with some previous studies (Liu et al., 2023; Wei et al., 2021) and underscore the importance of its alterations during this trimester in the occurrence of GDM. *Firmicutes* levels also rose in the GDM group during the third trimester (Li G. et al., 2021). Additional, specific *Firmicutes* phyla such as *Collinsella*, *Olsenella*, and *Clostridium* were significantly elevated in postpartum women diagnosed with GDM (Crusell et al., 2018). However, the phylum *Firmicutes* and class *Clostridia* were notably decreased in men with type 2 diabetes (T2DM) (Larsen et al., 2010). The variations in subject demographics and types of diabetes might account for these discrepancies. Regarding *Fusobacteria*, Wang et al.’s study (Wang et al., 2018) indicated an increase in women with GDM and a negative correlation between the *Faecalibacterium*/*Fusobacterium* ratio and GDM, aligning with Cortez et al.’s findings during late pregnancy in GDM women (Cortez et al., 2019). Significantly, a higher *Faecalibacterium*/*Fusobacterium* ratio has been linked to obesity-induced low-grade inflammation (Roselli et al., 2017). Our findings suggest that changes in intestinal flora structure during the second trimester are closely related to the development of GDM.

To mitigate the impact of population background factors and gestational age on microbiota analyses, we utilized the indices of the variance of relative abundance (VRA) and the ratios of relative abundance (RRA). The RRA values revealed substantial increases or decreases in dominant microbiota from the first to the second trimester in the GDM group, after adjusting for background changes. Specifically, the RRAs of phyla and genera associated with inflammation, such as *Staphylococcus*, *Streptococcus*, *Enterococcus*, and *Collinsella*, exceeded the cut-off value of 1.5, indicating a positive correlation with GDM. These findings are consistent with previous studies reporting increased levels of inflammatory bacteria in GDM patients (Wei et al., 2021; Vavreckova et al., 2022; Rold et al., 2022) and suggest that inflammation may play a crucial role in the gut microbiota changes associated with GDM. *Staphylococcus*, *Streptococcus*, and *Enterococcus* are identified as opportunistic pathogens (Mao et al., 2024), which are linked to intestinal inflammation. Pathogenic microbiotas may trigger Toll-like receptor 4 via lipopolysaccharides (LPS) in the gut mucosal barrier, contributing to metabolic inflammation (Tang et al., 2021). Inflammation-associated bacteria such as *Collinsella* and *Streptococcus* were markedly elevated not only in individuals with T2DM (Zhong et al., 2019; Takagi et al., 2020) but also in those with GDM (Hasain et al., 2020). *Collinsella* is recognized for its pro-inflammatory influence during pregnancy (Gomez-Arango et al., 2018) and has been positively linked to elevated maternal insulin levels (Gomez-Arango et al., 2016, 2018). Furthermore, our study observed that the relative abundance of *Bifidobacterium_388775* was below 0.8 in the GDM group, aligning with previous research findings (Chen F. et al., 2021). *Bifidobacterium* species are renowned for their anti-inflammatory properties in neonatal diabetes (Alsharairi, 2023) and acute pancreatitis (Li et al., 2022). Supplementation with *Bifidobacterium* probiotics helps mitigate inflammation and oxidative stress in GDM (Zhang et al., 2019). T2DM patients typically have reduced numbers of *Bifidobacterium* species (Lê et al., 2013). Consequently, the results of this study might indicate a future risk of developing T2DM in these individuals and could therefore be pivotal for preventing T2DM following GDM.

PICRUSt2 analysis predicted the upregulation of inflammatory pathways in the second trimester within the GDM group in our study, further supporting the link between gut microbiota dysbiosis, inflammation, and GDM. It has been reported that the low-grade inflammation caused by gut microbiota dysbiosis may contribute to insulin resistance and hyperglycemia in women with GDM (Hu et al., 2023). These results suggest that inflammation may play a role in the gut microbiota changes associated with GDM. Nevertheless, we did not detect any clinically apparent inflammation in the GDM group (Supplementary Table S12). We attribute this finding to two potential reasons. Firstly, the relatively small sample size of the GDM group might not have been sufficient to demonstrate a significant statistical difference. Secondly, based on findings from other studies (Hasain et al., 2020), the inflammation associated with GDM is often considered low-grade, which may explain the absence of a typical inflammatory response.

Stratified analysis by BMI revealed distinct microbiota changes in overweight or obese women with GDM compared to normal-weight women. Specifically, inflammation-associated genera like *Streptococcus*, *Enterocloster*, *Collinsella*, *Gemmiger_A_73129* and *Enterocloster* increased, while the beneficial butyrate-producer Megamonas decreased in overweight or obese GDM women. These findings, along with the upregulation of inflammatory pathways predicted by PICRUSt2 analysis, suggest that inflammatory microbiota and pathways may play a significant role in the development of GDM in this subset of the population. Higher *Gemmiger* levels correlate with inflammation (Lang et al., 2021), *Enterocloster* is linked to frailty and inflammatory markers (Dong et al., 2024), and lower *Megamonas* levels are associated with systemic inflammation (Chen Yan et al., 2024). Similarly, the *colostrum* of women who are obese or have GDM showed an overabundance of *Staphylococcus* compared to the control group (Gámez-Valdez et al., 2021). A metabolomic study found elevated levels of branched-chain amino acids and glycoprotein acetylation, a marker of low-grade inflammation, in overweight or obese women with GDM (Mokkala et al., 2020). BMI critically mediates the link between systemic inflammation and diabetes (Chen Yongze et al., 2024). These findings suggest that inflammatory microbiota and pathways may play a significant role in GDM development in this group, warranting further research.

This study has several limitations. Firstly, the incidence of GDM in the enrolled population was lower than expected, resulting in a limited sample size for the GDM group. This may have reduced the statistical power of group comparisons and led to missed detection of significantly different bacterial flora. Secondly, information on diet, exercise, and other potential confounders was not collected, which may have influenced gut microbiota composition. However, the longitudinal comparison from the first to the second trimester helped mitigate these confounding effects. Thirdly, the 16S rRNA sequencing technology used in this study has inherent limitations and may not fully capture the genetic information of bacterial flora. Further investigation using metagenomic and metabolomic analyses is needed to reveal the specific functions and metabolic pathways of different bacterial communities.

## Conclusion

In conclusion, this nested cohort study investigated the dynamics of gut microbiota from the first to the second trimester before GDM diagnosis and uncovered significant alterations in the GDM cohort compared to the control group. The key bacteria abundant in the GDM group, such as phyla *Fusobacteriota*, *Firmicutes_D*, and genus *Fusobacterium_A*, showed notable increases between the first and second trimesters. Additionally, there was an escalation in microbiota associated with inflammation, such as *Staphylococcus*, *Streptococcus*, and *Enterocloster*, in GDM patients, particularly among overweight or obese individuals. These findings highlight the intricate link between disruptions in gut microbiota and GDM, suggesting that elevated inflammatory responses related to microbiota dysbiosis in overweight or obese women during this critical period may influence GDM development. Further research into the role of inflammatory responses driven by gut microbiota in GDM among obese women is essential to elucidate the underlying mechanisms and develop targeted interventions.

## Data Availability

The original contributions presented in the study are publicly available. This data can be found in the NCBI repository: https://www.ncbi.nlm.nih.gov/bioproject/?term=PRJNA1193797 and the sequencing data: https://www.ncbi.nlm.nih.gov/sra/?term=SRP549371.

## Footnotes

^1^ https://docs.qiime2.org/2019.1/

^2^ https://greengenes2.ucsd.edu/
