# Supplementary material for: Dynamic changes of gut microbiota between the first and second trimester for women with gestational diabetes mellitus and their correlations with BMI: a nested cohort study in China
Source: Front Microbiol. 2024 Dec 11;15:1467414. doi: 10.3389/fmicb.2024.1467414 (PMC11669307; doi:10.3389/fmicb.2024.1467414)
Supplement: Supplementary file 1 [file Table_1.DOCX]

Table S1 The predominant phyla in the first and the second trimester

| OTU | mean of RA in total | mean of RA in FT | mean of RA in ST |
| --- | --- | --- | --- |
| Bacteroidota | 43.589% | 47.749% | 39.429% |
| Firmicutes_A | 36.283% | 34.532% | 38.034% |
| Proteobacteria | 7.190% | 5.266% | 9.113% |
| Firmicutes_C | 5.371% | 5.295% | 5.446% |
| Actinobacteriota | 2.975% | 2.604% | 3.345% |
| Firmicutes_D | 2.831% | 2.429% | 3.232% |
| Desulfobacterota_I | 0.337% | 0.290% | 0.383% |
| Verrucomicrobiota | 0.684% | 1.091% | 0.276% |
| Fusobacteriota | 0.423% | 0.592% | 0.253% |
| Chloroflexota | 0.037% | 0.003% | 0.072% |
| Other | 0.039% | 0.007% | 0.071% |
| Acidobacteriota | 0.036% | 0.002% | 0.070% |
| unclassified | 0.053% | 0.044% | 0.062% |
| Cyanobacteria | 0.030% | 0.014% | 0.046% |
| Thermoproteota | 0.018% | 0.000% | 0.036% |
| Planctomycetota | 0.014% | 0.000% | 0.028% |
| Thermoplasmatota | 0.013% | 0.001% | 0.026% |
| Deinococcota | 0.014% | 0.004% | 0.024% |
| Firmicutes_B_370539 | 0.020% | 0.020% | 0.021% |
| Gemmatimonadota | 0.008% | 0.001% | 0.016% |
| Synergistota | 0.036% | 0.056% | 0.016% |

RA: relative abundance

Table S2 The predominant genera in the first and the second trimester

| OTU | mean of RA in total | mean of RA in FT | mean of RA in ST |
| --- | --- | --- | --- |
| Phocaeicola_A_858004 | 15.996% | 17.871% | 14.121% |
| Prevotella | 12.113% | 11.611% | 12.615% |
| Faecalibacterium | 7.773% | 8.277% | 7.269% |
| Gemmiger_A_73129 | 3.679% | 2.088% | 5.270% |
| Bacteroides_H | 6.543% | 8.068% | 5.018% |
| Escherichia_710834 | 3.463% | 2.821% | 4.105% |
| Lachnospira | 2.873% | 2.622% | 3.124% |
| Acinetobacter | 1.960% | 1.200% | 2.719% |
| Bifidobacterium_388775 | 2.157% | 2.048% | 2.267% |
| Blautia_A_141781 | 1.971% | 1.765% | 2.176% |
| Collinsella | 1.424% | 0.933% | 1.915% |
| unclassified | 1.787% | 1.838% | 1.736% |
| Enterocloster | 1.047% | 0.756% | 1.339% |
| Phascolarctobacterium_A | 1.268% | 1.235% | 1.301% |
| Fusicatenibacter | 1.146% | 1.006% | 1.286% |
| Holdemanella | 0.856% | 0.461% | 1.251% |
| Anaerostipes | 1.152% | 1.057% | 1.247% |
| Streptococcus | 1.138% | 1.062% | 1.215% |
| Staphylococcus | 0.592% | 0.034% | 1.150% |
| Agathobacter_164117 | 1.389% | 1.652% | 1.126% |
| Faecalibacillus | 0.826% | 0.594% | 1.058% |
| Klebsiella_724518 | 1.034% | 1.034% | 1.034% |
| Roseburia | 1.039% | 1.059% | 1.019% |
| Mediterraneibacter_A_155507 | 0.823% | 0.643% | 1.003% |
| Dialister | 1.224% | 1.491% | 0.957% |
| Parabacteroides_B_862066 | 1.258% | 1.590% | 0.926% |
| Megamonas | 1.492% | 2.083% | 0.902% |
| Acetatifactor | 1.041% | 1.318% | 0.765% |

RA: relative abundance

Table S3 Comparison of the relative abundance of the gut microbiota between the GDM and ND group in the participants with normal weight in the first trimester

| OTU | NW_GDM_FT | NW_ND_FT | Test-Statistic | P |
| --- | --- | --- | --- | --- |
| Escherichia_710834 | 3.30% | 1.16% | 7.621 | 0.006 |
| Oxalobacter_566322 | 0.01% | 0.00% | 6.846 | 0.009 |
| Megasphaera_A_38685 | 0.00% | 0.05% | 5.675 | 0.017 |
| Massilia | 0.02% | 0.01% | 5.056 | 0.025 |
| Campylobacter_B | 0.01% | 0.00% | 4.896 | 0.027 |
| Thermus_A | 0.01% | 0.00% | 4.586 | 0.032 |
| Slackia_A | 0.00% | 0.00% | 4.270 | 0.039 |
| UBA3402 | 0.03% | 0.18% | 4.002 | 0.045 |
| Acetatifactor | 0.26% | 1.10% | 3.993 | 0.046 |
| Blautia_A_141780 | 0.11% | 0.06% | 3.911 | 0.048 |

Table S4 Comparison of the relative abundance of the gut microbiota between the GDM and ND group in the participants with overweight or obese in the first trimester

| OTU | OW_GDM_FT | OW_ND_FT | Test-Statistic | P |
| --- | --- | --- | --- | --- |
| Vescimonas | 0.122% | 0.229% | 4.640 | 0.031 |
| UBA1417 | 0.015% | 0.064% | 5.936 | 0.015 |
| CAG_510 | 0.000% | 0.006% | 4.235 | 0.040 |
| Gordonibacter | 0.000% | 0.005% | 4.235 | 0.040 |
| Brevundimonas | 0.000% | 0.004% | 4.235 | 0.040 |
| Eubacterium_O_258270 | 0.000% | 0.003% | 4.235 | 0.040 |
| Scatomonas | 0.000% | 0.003% | 3.973 | 0.046 |
| Haemophilus_D_735815 | 0.136% | 0.015% | 4.246 | 0.039 |

Table S5 Comparison of the relative abundance of the gut microbiota between the GDM and ND group in the participants with normal weight in the second trimester

| OTU | NW_GDM_ST | NW_ND_ST | Test-Statistic | P |
| --- | --- | --- | --- | --- |
| Christensenella | 0.0026% | 0.0028% | 5.030 | 0.025 |
| Coprenecus | 0.0012% | 0.0034% | 4.138 | 0.042 |
| Intestinibacter | 0.0727% | 0.0879% | 4.099 | 0.043 |

Table S6 Comparison of the relative abundance of the gut microbiota between the GDM and ND group in the participants with overweight or obese in the second trimester

| OTU | OW_GDM_ST | OW_ND_ST | Test-Statistic | P |
| --- | --- | --- | --- | --- |
| Enterocloster | 1.997% | 0.457% | 4.246 | 0.039 |
| Clostridium_Q_135822 | 0.661% | 0.254% | 5.482 | 0.019 |
| Adlercreutzia_404257 | 0.121% | 0.014% | 4.660 | 0.031 |
| Dielma | 0.000% | 0.078% | 4.032 | 0.045 |
| Phocaeicola_A_858004 | 5.564% | 13.695% | 3.868 | 0.049 |
| Bacteroides_H | 2.232% | 11.579% | 4.246 | 0.039 |

Table S7 Matched intergroup comparisons of the relative abundance within GDM group

| microbiota | In ND group | | | |  | In GDM group | | | |
| --- | --- | --- | --- | --- | --- | --- | --- | --- | --- |
|  | Mean AR in FT | Mean AR in ST | Z | P |  | Mean AR in the FT | Mean AR in the ST | Z | P |
| CAG_41 | 0.172% | 0.281% | 2.494 | 0.013 |  | 0.196% | 0.421% | 2.845 | 0.004 |
| Adlercreutzia_404257 | 0.033% | 0.026% | -1.417 | 0.157 |  | 0.031% | 0.071% | 2.756 | 0.006 |
| Fournierella | 0.004% | 0.009% | 2.844 | 0.004 |  | 0.000% | 0.010% | 2.521 | 0.012 |
| Eubacterium_R | 0.135% | 0.147% | 0.155 | 0.877 |  | 0.070% | 0.226% | 2.490 | 0.013 |
| UMGS1375 | 0.078% | 0.108% | 1.546 | 0.122 |  | 0.113% | 0.176% | 2.401 | 0.016 |
| Intestinibacter | 0.063% | 0.077% | 3.431 | 0.001 |  | 0.086% | 0.156% | 2.395 | 0.017 |
| Angelakisella | 0.043% | 0.027% | -1.003 | 0.316 |  | 0.043% | 0.006% | -2.366 | 0.018 |
| Burkholderia | 0.042% | 0.144% | 2.995 | 0.003 |  | 0.002% | 0.027% | 2.366 | 0.018 |
| Pseudoflavonifractor_80958 | 0.005% | 0.007% | 2.128 | 0.033 |  | 0.001% | 0.008% | 2.366 | 0.018 |
| Clostridium_T | 0.049% | 0.227% | 3.746 | 0.000 |  | 0.029% | 0.172% | 2.312 | 0.021 |
| Holdemania | 0.016% | 0.028% | 3.573 | 0.000 |  | 0.015% | 0.038% | 2.223 | 0.026 |
| Schaedlerella | 0.019% | 0.030% | 1.274 | 0.203 |  | 0.010% | 0.027% | 2.100 | 0.036 |
| Streptococcus | 0.521% | 1.166% | 3.802 | 0.000 |  | 0.288% | 1.308% | 2.045 | 0.041 |
| Pedobacter_887417 | 0.008% | 0.042% | 0.688 | 0.492 |  | 0.001% | 0.005% | 2.023 | 0.043 |
| Caecibacter | 0.006% | 0.016% | 1.234 | 0.217 |  | 0.008% | 0.030% | 1.992 | 0.046 |
| Fusobacterium_C | 0.004% | 0.021% | 1.338 | 0.181 |  | 0.002% | 0.014% | 1.992 | 0.046 |
| Oxalobacter_566322 | 0.002% | 0.002% | 0.745 | 0.456 |  | 0.004% | 0.001% | -1.992 | 0.046 |

Table S8 The comparison of VRA of the differentiated genera between the ND and GDM group

| genera | VRA in DN group (mean±SD) | VRA in GDM group (mean±SD) | P |
| --- | --- | --- | --- |
| Eubacterium_R | 0.012%±0.331% | 0.156%±0.3% | 0.022 |
| Allisonella | -0.002%±0.037% | 0.048%±0.146% | 0.036 |
| Fournierella | 0.006%±0.036% | 0.01%±0.011% | 0.017 |
| Intestinibacter | 0.014%±0.178% | 0.07%±0.077% | 0.027 |
| CAG_41 | 0.108%±0.466% | 0.225%±0.225% | 0.008 |
| Angelakisella | -0.016%±0.093% | -0.037%±0.082% | 0.032 |

RA: relative abundance

Table S9 The mean RA and RRA of the predominant phyla

| phyla | RA in GDM_FT group (mean±SD) | RA in GDM_ST group (mean±SD) | RA in ND_FT group (mean±SD) | RA in ND_ST group (mean±SD) | RRA^*^ |
| --- | --- | --- | --- | --- | --- |
| Bacteroidota | 50.105%(±31.235%) | 32.094%(±20.416%) | 47.433%(±20.132%) | 40.413%(±20.145%) | 0.752 |
| Actinobacteriota | 3.869%(±5.371%) | 4.751%(±6.235%) | 2.434%(±5.930%) | 3.157%(±6.525%) | 0.947 |
| Proteobacteria | 6.302%(±7.287%) | 10.680%(±9.602%) | 5.127%(±6.553%) | 8.903%(±11.814%) | 0.976 |
| Firmicutes_C | 3.187%(±1.967%) | 3.320%(±1.922%) | 5.577%(±7.687%) | 5.732%(±8.587%) | 1.014 |
| Firmicutes_D | 4.195%(±6.153%) | 6.346%(±6.408%) | 2.192%(±4.163%) | 2.814%(±4.357%) | 1.179 |
| Firmicutes_A | 31.908%(±18.852%) | 41.413%(±18.610%) | 34.884%(±15.980%) | 37.581%(±16.755%) | 1.205 |
| Desulfobacterota_I | 0.195%(±0.196%) | 0.452%(±0.433%) | 0.303%(±0.321%) | 0.374%(±0.348%) | 1.879 |
| Verrucomicrobiota | 0.145%(±0.228%) | 0.156%(±0.319%) | 1.218%(±6.653%) | 0.292%(±0.906%) | 4.503 |
| Fusobacteriota | 0.044%(±0.038%) | 0.525%(±1.061%) | 0.665%(±3.217%) | 0.217%(±0.987%) | 36.223 |

^*^RRA: the ratio of the relative abundance, RRA= (RA^GDM_ST^ / RA^GDM_FT^ ) / (RA^ND_ST^ / RA^ND_FT^); RA: relative abundance

Table S10 The mean RA and RRA of the significant genera

| genera | RA in GDM_FT group (mean±SD) | RA in GDM_ST group (mean±SD) | RA in ND_FT group (mean±SD) | RA in ND_ST group (mean±SD) | RRA |
| --- | --- | --- | --- | --- | --- |
| Bifidobacterium_388775 | 2.827%(±4.061%) | 2.351%(±2.625%) | 1.639%(±5.043%) | 1.944%(±5.563%) | 0.701 |
| Phocaeicola_A_858004 | 19.365%(±24.330%) | 13.483%(±12.444%) | 18.872%(±15.444%) | 17.671%(±14.212%) | 0.744 |
| Faecalibacillus | 0.738%(±1.502%) | 1.122%(±1.289%) | 0.577%(±2.113%) | 0.575%(±0.971%) | 1.526 |
| Collinsella | 0.850%(±1.303%) | 2.085%(±3.900%) | 0.649%(±1.767%) | 0.944%(±2.275%) | 1.686 |
| Megamonas | 0.450%(±0.414%) | 0.675%(±0.578%) | 2.592%(±7.196%) | 2.302%(±7.350%) | 1.688 |
| Mediterraneibacter_A_155507 | 0.562%(±0.485%) | 1.050%(±1.589%) | 0.652%(±0.869%) | 0.654%(±0.695%) | 1.862 |
| Enterocloster | 0.754%(±0.729%) | 1.410%(±2.127%) | 0.812%(±0.856%) | 0.756%(±0.665%) | 2.007 |
| Streptococcus | 0.288%(±0.260%) | 1.308%(±1.956%) | 0.521%(±1.575%) | 1.166%(±3.695%) | 2.028 |
| CAG_269 | 0.375%(±0.427%) | 1.025%(±2.631%) | 0.455%(±1.293%) | 0.278%(±0.768%) | 4.481 |
| Staphylococcus | 0.017%(±0.028%) | 1.303%(±4.247%) | 0.011%(±0.057%) | 0.036%(±0.085%) | 23.722 |

^*^RRA: the ratio of the relative abundance, RRA= (RA^GDM_ST^ / RA^GDM_FT^ ) / (RA^ND_ST^ / RA^ND_FT^); RA: relative abundance

Table S11 The comparison results of the KEGG pathways between the GDM and ND group in the participants with normal weight in the second trimester

| KEGG pathways | p |
| --- | --- |
| Human Diseases;Cancer: overview;Central carbon metabolism in cancer | 0.856 |
| Human Diseases;Cancer: overview;Chemical carcinogenesis | 0.471 |
| Human Diseases;Cancer: overview;Choline metabolism in cancer | 0.826 |
| Human Diseases;Cancer: overview;MicroRNAs in cancer | 0.770 |
| Human Diseases;Cancer: overview;Pathways in cancer | 0.879 |
| Human Diseases;Cancer: overview;Proteoglycans in cancer | 0.867 |
| Human Diseases;Cancer: overview;Viral carcinogenesis | 0.972 |
| Human Diseases;Cancer: specific types;Bladder cancer | 0.368 |
| Human Diseases;Cancer: specific types;Colorectal cancer | 0.525 |
| Human Diseases;Cancer: specific types;Gastric cancer | 0.816 |
| Human Diseases;Cancer: specific types;Hepatocellular carcinoma | 0.592 |
| Human Diseases;Cancer: specific types;Prostate cancer | 0.833 |
| Human Diseases;Cancer: specific types;Renal cell carcinoma | 0.745 |
| Human Diseases;Cancer: specific types;Small cell lung cancer | 0.524 |
| Human Diseases;Cardiovascular disease;Arrhythmogenic right ventricular cardiomyopathy (ARVC) | 0.362 |
| Human Diseases;Cardiovascular disease;Dilated cardiomyopathy (DCM) | 0.369 |
| Human Diseases;Cardiovascular disease;Fluid shear stress and atherosclerosis | 0.815 |
| Human Diseases;Cardiovascular disease;Hypertrophic cardiomyopathy (HCM) | 0.510 |
| Human Diseases;Cardiovascular disease;Viral myocarditis | 0.524 |
| Human Diseases;Drug resistance: antimicrobial;Cationic antimicrobial peptide (CAMP) resistance | 0.473 |
| Human Diseases;Drug resistance: antimicrobial;Vancomycin resistance | 0.977 |
| Human Diseases;Drug resistance: antimicrobial;beta-Lactam resistance | 0.883 |
| Human Diseases;Drug resistance: antineoplastic;Antifolate resistance | 0.819 |
| Human Diseases;Drug resistance: antineoplastic;Platinum drug resistance | 0.872 |
| Human Diseases;Endocrine and metabolic disease;Cushing syndrome | 0.745 |
| Human Diseases;Endocrine and metabolic disease;Insulin resistance | 0.912 |
| Human Diseases;Endocrine and metabolic disease;Non-alcoholic fatty liver disease (NAFLD) | 0.401 |
| Human Diseases;Endocrine and metabolic disease;Type I diabetes mellitus | 0.935 |
| Human Diseases;Endocrine and metabolic disease;Type II diabetes mellitus | 0.825 |
| Human Diseases;Immune disease;Primary immunodeficiency | 0.873 |
| Human Diseases;Immune disease;Systemic lupus erythematosus | 0.738 |
| Human Diseases;Infectious disease: bacterial;Bacterial invasion of epithelial cells | 0.279 |
| Human Diseases;Infectious disease: bacterial;Epithelial cell signaling in Helicobacter pylori infection | 0.911 |
| Human Diseases;Infectious disease: bacterial;Legionellosis | 0.799 |
| Human Diseases;Infectious disease: bacterial;Pathogenic Escherichia coli infection | 0.746 |
| Human Diseases;Infectious disease: bacterial;Pertussis | 0.292 |
| Human Diseases;Infectious disease: bacterial;Salmonella infection | 0.558 |
| Human Diseases;Infectious disease: bacterial;Shigellosis | 0.860 |
| Human Diseases;Infectious disease: bacterial;Staphylococcus aureus infection | 0.746 |
| Human Diseases;Infectious disease: bacterial;Tuberculosis | 0.539 |
| Human Diseases;Infectious disease: bacterial;Vibrio cholerae infection | 0.628 |
| Human Diseases;Infectious disease: parasitic;African trypanosomiasis | 0.403 |
| Human Diseases;Infectious disease: parasitic;Amoebiasis | 0.528 |
| Human Diseases;Infectious disease: parasitic;Chagas disease (American trypanosomiasis) | 0.351 |
| Human Diseases;Infectious disease: parasitic;Toxoplasmosis | 0.525 |
| Human Diseases;Infectious disease: viral;Hepatitis B | 0.511 |
| Human Diseases;Infectious disease: viral;Herpes simplex virus 1 infection | 0.505 |
| Human Diseases;Infectious disease: viral;Human cytomegalovirus infection | 0.525 |
| Human Diseases;Infectious disease: viral;Human papillomavirus infection | 0.927 |
| Human Diseases;Infectious disease: viral;Influenza A | 0.513 |
| Human Diseases;Infectious disease: viral;Kaposi sarcoma-associated herpesvirus infection | 0.525 |
| Human Diseases;Neurodegenerative disease;Alzheimer disease | 0.694 |
| Human Diseases;Neurodegenerative disease;Amyotrophic lateral sclerosis (ALS) | 0.480 |
| Human Diseases;Neurodegenerative disease;Huntington disease | 0.999 |
| Human Diseases;Neurodegenerative disease;Parkinson disease | 0.461 |
| Human Diseases;Neurodegenerative disease;Prion diseases | 0.242 |
| Human Diseases;Substance dependence;Alcoholism | 0.837 |
| Human Diseases;Substance dependence;Amphetamine addiction | 0.852 |
| Human Diseases;Substance dependence;Cocaine addiction | 0.856 |
| Metabolism;Amino acid metabolism;Alanine, aspartate and glutamate metabolism | 0.868 |
| Metabolism;Amino acid metabolism;Arginine and proline metabolism | 0.313 |
| Metabolism;Amino acid metabolism;Arginine biosynthesis | 0.616 |
| Metabolism;Amino acid metabolism;Cysteine and methionine metabolism | 0.739 |
| Metabolism;Amino acid metabolism;Glycine, serine and threonine metabolism | 0.594 |
| Metabolism;Amino acid metabolism;Histidine metabolism | 0.355 |
| Metabolism;Amino acid metabolism;Lysine biosynthesis | 0.756 |
| Metabolism;Amino acid metabolism;Lysine degradation | 0.903 |
| Metabolism;Amino acid metabolism;Phenylalanine metabolism | 0.920 |
| Metabolism;Amino acid metabolism;Phenylalanine, tyrosine and tryptophan biosynthesis | 0.730 |
| Metabolism;Amino acid metabolism;Tryptophan metabolism | 0.967 |
| Metabolism;Amino acid metabolism;Tyrosine metabolism | 0.791 |
| Metabolism;Amino acid metabolism;Valine, leucine and isoleucine biosynthesis | 0.831 |
| Metabolism;Amino acid metabolism;Valine, leucine and isoleucine degradation | 0.536 |
| Metabolism;Biosynthesis of other secondary metabolites;Acarbose and validamycin biosynthesis | 0.601 |
| Metabolism;Biosynthesis of other secondary metabolites;Betalain biosynthesis | 0.469 |
| Metabolism;Biosynthesis of other secondary metabolites;Biosynthesis of various secondary metabolites - part 1 | 0.685 |
| Metabolism;Biosynthesis of other secondary metabolites;Carbapenem biosynthesis | 0.923 |
| Metabolism;Biosynthesis of other secondary metabolites;Flavone and flavonol biosynthesis | 0.738 |
| Metabolism;Biosynthesis of other secondary metabolites;Flavonoid biosynthesis | 0.281 |
| Metabolism;Biosynthesis of other secondary metabolites;Glucosinolate biosynthesis | 0.639 |
| Metabolism;Biosynthesis of other secondary metabolites;Isoquinoline alkaloid biosynthesis | 0.654 |
| Metabolism;Biosynthesis of other secondary metabolites;Monobactam biosynthesis | 0.914 |
| Metabolism;Biosynthesis of other secondary metabolites;Neomycin, kanamycin and gentamicin biosynthesis | 0.598 |
| Metabolism;Biosynthesis of other secondary metabolites;Novobiocin biosynthesis | 0.806 |
| Metabolism;Biosynthesis of other secondary metabolites;Penicillin and cephalosporin biosynthesis | 0.925 |
| Metabolism;Biosynthesis of other secondary metabolites;Phenazine biosynthesis | 0.685 |
| Metabolism;Biosynthesis of other secondary metabolites;Phenylpropanoid biosynthesis | 0.906 |
| Metabolism;Biosynthesis of other secondary metabolites;Prodigiosin biosynthesis | 0.943 |
| Metabolism;Biosynthesis of other secondary metabolites;Staurosporine biosynthesis | 0.800 |
| Metabolism;Biosynthesis of other secondary metabolites;Stilbenoid, diarylheptanoid and gingerol biosynthesis | 0.284 |
| Metabolism;Biosynthesis of other secondary metabolites;Streptomycin biosynthesis | 0.595 |
| Metabolism;Biosynthesis of other secondary metabolites;Tropane, piperidine and pyridine alkaloid biosynthesis | 0.575 |
| Metabolism;Carbohydrate metabolism;Amino sugar and nucleotide sugar metabolism | 0.933 |
| Metabolism;Carbohydrate metabolism;Ascorbate and aldarate metabolism | 0.759 |
| Metabolism;Carbohydrate metabolism;Butanoate metabolism | 0.870 |
| Metabolism;Carbohydrate metabolism;C5-Branched dibasic acid metabolism | 0.736 |
| Metabolism;Carbohydrate metabolism;Citrate cycle (TCA cycle) | 0.881 |
| Metabolism;Carbohydrate metabolism;Fructose and mannose metabolism | 0.749 |
| Metabolism;Carbohydrate metabolism;Galactose metabolism | 0.849 |
| Metabolism;Carbohydrate metabolism;Glycolysis / Gluconeogenesis | 0.907 |
| Metabolism;Carbohydrate metabolism;Glyoxylate and dicarboxylate metabolism | 0.865 |
| Metabolism;Carbohydrate metabolism;Inositol phosphate metabolism | 0.382 |
| Metabolism;Carbohydrate metabolism;Pentose and glucuronate interconversions | 0.419 |
| Metabolism;Carbohydrate metabolism;Pentose phosphate pathway | 0.760 |
| Metabolism;Carbohydrate metabolism;Propanoate metabolism | 0.809 |
| Metabolism;Carbohydrate metabolism;Pyruvate metabolism | 0.837 |
| Metabolism;Carbohydrate metabolism;Starch and sucrose metabolism | 0.999 |
| Metabolism;Chemical structure transformation maps;Biosynthesis of terpenoids and steroids | 0.642 |
| Metabolism;Energy metabolism;Carbon fixation in photosynthetic organisms | 0.854 |
| Metabolism;Energy metabolism;Carbon fixation pathways in prokaryotes | 0.977 |
| Metabolism;Energy metabolism;Methane metabolism | 0.887 |
| Metabolism;Energy metabolism;Nitrogen metabolism | 0.542 |
| Metabolism;Energy metabolism;Oxidative phosphorylation | 0.902 |
| Metabolism;Energy metabolism;Photosynthesis | 0.943 |
| Metabolism;Energy metabolism;Sulfur metabolism | 0.663 |
| Metabolism;Global and overview maps;2-Oxocarboxylic acid metabolism | 0.739 |
| Metabolism;Global and overview maps;Biosynthesis of amino acids | 0.759 |
| Metabolism;Global and overview maps;Biosynthesis of secondary metabolites | 0.791 |
| Metabolism;Global and overview maps;Carbon metabolism | 0.385 |
| Metabolism;Global and overview maps;Degradation of aromatic compounds | 0.712 |
| Metabolism;Global and overview maps;Fatty acid metabolism | 0.719 |
| Metabolism;Global and overview maps;Metabolic pathways | 0.992 |
| Metabolism;Global and overview maps;Microbial metabolism in diverse environments | 0.856 |
| Metabolism;Glycan biosynthesis and metabolism;Glycosaminoglycan degradation | 0.786 |
| Metabolism;Glycan biosynthesis and metabolism;Glycosphingolipid biosynthesis - ganglio series | 0.598 |
| Metabolism;Glycan biosynthesis and metabolism;Glycosphingolipid biosynthesis - globo and isoglobo series | 0.608 |
| Metabolism;Glycan biosynthesis and metabolism;Glycosphingolipid biosynthesis - lacto and neolacto series | 0.444 |
| Metabolism;Glycan biosynthesis and metabolism;Lipoarabinomannan (LAM) biosynthesis | 0.630 |
| Metabolism;Glycan biosynthesis and metabolism;Lipopolysaccharide biosynthesis | 0.539 |
| Metabolism;Glycan biosynthesis and metabolism;Mannose type O-glycan biosynthesis | 0.225 |
| Metabolism;Glycan biosynthesis and metabolism;N-Glycan biosynthesis | 0.924 |
| Metabolism;Glycan biosynthesis and metabolism;Other glycan degradation | 0.613 |
| Metabolism;Glycan biosynthesis and metabolism;Other types of O-glycan biosynthesis | 0.228 |
| Metabolism;Glycan biosynthesis and metabolism;Peptidoglycan biosynthesis | 0.894 |
| Metabolism;Glycan biosynthesis and metabolism;Various types of N-glycan biosynthesis | 0.600 |
| Metabolism;Lipid metabolism;Arachidonic acid metabolism | 0.508 |
| Metabolism;Lipid metabolism;Biosynthesis of unsaturated fatty acids | 0.920 |
| Metabolism;Lipid metabolism;Ether lipid metabolism | 0.668 |
| Metabolism;Lipid metabolism;Fatty acid biosynthesis | 0.778 |
| Metabolism;Lipid metabolism;Fatty acid degradation | 0.913 |
| Metabolism;Lipid metabolism;Glycerolipid metabolism | 0.785 |
| Metabolism;Lipid metabolism;Glycerophospholipid metabolism | 0.615 |
| Metabolism;Lipid metabolism;Linoleic acid metabolism | 0.603 |
| Metabolism;Lipid metabolism;Primary bile acid biosynthesis | 0.737 |
| Metabolism;Lipid metabolism;Secondary bile acid biosynthesis | 0.713 |
| Metabolism;Lipid metabolism;Sphingolipid metabolism | 0.713 |
| Metabolism;Lipid metabolism;Steroid biosynthesis | 0.918 |
| Metabolism;Lipid metabolism;Steroid hormone biosynthesis | 0.817 |
| Metabolism;Lipid metabolism;Synthesis and degradation of ketone bodies | 0.931 |
| Metabolism;Lipid metabolism;alpha-Linolenic acid metabolism | 0.407 |
| Metabolism;Metabolism of cofactors and vitamins;Biotin metabolism | 0.562 |
| Metabolism;Metabolism of cofactors and vitamins;Folate biosynthesis | 0.624 |
| Metabolism;Metabolism of cofactors and vitamins;Lipoic acid metabolism | 0.744 |
| Metabolism;Metabolism of cofactors and vitamins;Nicotinate and nicotinamide metabolism | 0.362 |
| Metabolism;Metabolism of cofactors and vitamins;One carbon pool by folate | 0.948 |
| Metabolism;Metabolism of cofactors and vitamins;Pantothenate and CoA biosynthesis | 0.815 |
| Metabolism;Metabolism of cofactors and vitamins;Porphyrin and chlorophyll metabolism | 0.807 |
| Metabolism;Metabolism of cofactors and vitamins;Retinol metabolism | 0.687 |
| Metabolism;Metabolism of cofactors and vitamins;Riboflavin metabolism | 0.533 |
| Metabolism;Metabolism of cofactors and vitamins;Thiamine metabolism | 0.792 |
| Metabolism;Metabolism of cofactors and vitamins;Ubiquinone and other terpenoid-quinone biosynthesis | 0.515 |
| Metabolism;Metabolism of cofactors and vitamins;Vitamin B6 metabolism | 0.843 |
| Metabolism;Metabolism of other amino acids;Cyanoamino acid metabolism | 0.750 |
| Metabolism;Metabolism of other amino acids;D-Alanine metabolism | 0.943 |
| Metabolism;Metabolism of other amino acids;D-Arginine and D-ornithine metabolism | 0.938 |
| Metabolism;Metabolism of other amino acids;D-Glutamine and D-glutamate metabolism | 0.882 |
| Metabolism;Metabolism of other amino acids;Glutathione metabolism | 0.591 |
| Metabolism;Metabolism of other amino acids;Phosphonate and phosphinate metabolism | 0.604 |
| Metabolism;Metabolism of other amino acids;Selenocompound metabolism | 0.502 |
| Metabolism;Metabolism of other amino acids;Taurine and hypotaurine metabolism | 0.690 |
| Metabolism;Metabolism of other amino acids;beta-Alanine metabolism | 0.851 |
| Metabolism;Metabolism of terpenoids and polyketides;Biosynthesis of ansamycins | 0.810 |
| Metabolism;Metabolism of terpenoids and polyketides;Biosynthesis of siderophore group nonribosomal peptides | 0.279 |
| Metabolism;Metabolism of terpenoids and polyketides;Biosynthesis of vancomycin group antibiotics | 0.689 |
| Metabolism;Metabolism of terpenoids and polyketides;Carotenoid biosynthesis | 0.855 |
| Metabolism;Metabolism of terpenoids and polyketides;Geraniol degradation | 0.539 |
| Metabolism;Metabolism of terpenoids and polyketides;Insect hormone biosynthesis | 0.563 |
| Metabolism;Metabolism of terpenoids and polyketides;Limonene and pinene degradation | 0.977 |
| Metabolism;Metabolism of terpenoids and polyketides;Nonribosomal peptide structures | 0.609 |
| Metabolism;Metabolism of terpenoids and polyketides;Polyketide sugar unit biosynthesis | 0.696 |
| Metabolism;Metabolism of terpenoids and polyketides;Sesquiterpenoid and triterpenoid biosynthesis | 0.887 |
| Metabolism;Metabolism of terpenoids and polyketides;Terpenoid backbone biosynthesis | 0.866 |
| Metabolism;Metabolism of terpenoids and polyketides;Tetracycline biosynthesis | 0.476 |
| Metabolism;Metabolism of terpenoids and polyketides;Zeatin biosynthesis | 0.857 |
| Metabolism;Nucleotide metabolism;Purine metabolism | 0.731 |
| Metabolism;Nucleotide metabolism;Pyrimidine metabolism | 0.767 |
| Metabolism;Xenobiotics biodegradation and metabolism;Aminobenzoate degradation | 0.665 |
| Metabolism;Xenobiotics biodegradation and metabolism;Atrazine degradation | 0.410 |
| Metabolism;Xenobiotics biodegradation and metabolism;Benzoate degradation | 0.975 |
| Metabolism;Xenobiotics biodegradation and metabolism;Caprolactam degradation | 0.666 |
| Metabolism;Xenobiotics biodegradation and metabolism;Chloroalkane and chloroalkene degradation | 0.859 |
| Metabolism;Xenobiotics biodegradation and metabolism;Chlorocyclohexane and chlorobenzene degradation | 0.977 |
| Metabolism;Xenobiotics biodegradation and metabolism;Dioxin degradation | 0.956 |
| Metabolism;Xenobiotics biodegradation and metabolism;Drug metabolism - cytochrome P450 | 0.699 |
| Metabolism;Xenobiotics biodegradation and metabolism;Drug metabolism - other enzymes | 0.626 |
| Metabolism;Xenobiotics biodegradation and metabolism;Ethylbenzene degradation | 0.328 |
| Metabolism;Xenobiotics biodegradation and metabolism;Fluorobenzoate degradation | 0.565 |
| Metabolism;Xenobiotics biodegradation and metabolism;Furfural degradation | 0.496 |
| Metabolism;Xenobiotics biodegradation and metabolism;Metabolism of xenobiotics by cytochrome P450 | 0.687 |
| Metabolism;Xenobiotics biodegradation and metabolism;Naphthalene degradation | 0.487 |
| Metabolism;Xenobiotics biodegradation and metabolism;Nitrotoluene degradation | 0.829 |
| Metabolism;Xenobiotics biodegradation and metabolism;Polycyclic aromatic hydrocarbon degradation | 0.941 |
| Metabolism;Xenobiotics biodegradation and metabolism;Steroid degradation | 0.556 |
| Metabolism;Xenobiotics biodegradation and metabolism;Styrene degradation | 0.486 |
| Metabolism;Xenobiotics biodegradation and metabolism;Toluene degradation | 0.501 |
| Metabolism;Xenobiotics biodegradation and metabolism;Xylene degradation | 0.941 |
| Organismal Systems;Aging;Longevity regulating pathway | 0.888 |
| Organismal Systems;Aging;Longevity regulating pathway - multiple species | 0.477 |
| Organismal Systems;Aging;Longevity regulating pathway - worm | 0.441 |
| Organismal Systems;Circulatory system;Adrenergic signaling in cardiomyocytes | 0.368 |
| Organismal Systems;Circulatory system;Cardiac muscle contraction | 0.759 |
| Organismal Systems;Digestive system;Bile secretion | 0.603 |
| Organismal Systems;Digestive system;Carbohydrate digestion and absorption | 0.819 |
| Organismal Systems;Digestive system;Mineral absorption | 0.996 |
| Organismal Systems;Digestive system;Pancreatic secretion | 0.379 |
| Organismal Systems;Digestive system;Protein digestion and absorption | 0.452 |
| Organismal Systems;Digestive system;Salivary secretion | 0.609 |
| Organismal Systems;Endocrine system;Adipocytokine signaling pathway | 0.865 |
| Organismal Systems;Endocrine system;Estrogen signaling pathway | 0.834 |
| Organismal Systems;Endocrine system;Glucagon signaling pathway | 0.610 |
| Organismal Systems;Endocrine system;Insulin secretion | 0.369 |
| Organismal Systems;Endocrine system;Insulin signaling pathway | 0.634 |
| Organismal Systems;Endocrine system;Melanogenesis | 0.846 |
| Organismal Systems;Endocrine system;Oxytocin signaling pathway | 0.422 |
| Organismal Systems;Endocrine system;PPAR signaling pathway | 0.897 |
| Organismal Systems;Endocrine system;Progesterone-mediated oocyte maturation | 0.833 |
| Organismal Systems;Endocrine system;Prolactin signaling pathway | 0.844 |
| Organismal Systems;Endocrine system;Renin secretion | 0.991 |
| Organismal Systems;Endocrine system;Renin-angiotensin system | 0.884 |
| Organismal Systems;Endocrine system;Thyroid hormone signaling pathway | 0.864 |
| Organismal Systems;Endocrine system;Thyroid hormone synthesis | 0.559 |
| Organismal Systems;Environmental adaptation;Circadian entrainment | 0.365 |
| Organismal Systems;Environmental adaptation;Plant-pathogen interaction | 0.661 |
| Organismal Systems;Environmental adaptation;Thermogenesis | 0.950 |
| Organismal Systems;Excretory system;Proximal tubule bicarbonate reclamation | 0.924 |
| Organismal Systems;Immune system;Antigen processing and presentation | 0.833 |
| Organismal Systems;Immune system;Hematopoietic cell lineage | 0.650 |
| Organismal Systems;Immune system;IL-17 signaling pathway | 0.847 |
| Organismal Systems;Immune system;NOD-like receptor signaling pathway | 0.879 |
| Organismal Systems;Immune system;RIG-I-like receptor signaling pathway | 0.536 |
| Organismal Systems;Immune system;Th17 cell differentiation | 0.833 |
| Organismal Systems;Immune system;Toll and Imd signaling pathway | 0.415 |
| Organismal Systems;Nervous system;Dopaminergic synapse | 0.838 |
| Organismal Systems;Nervous system;GABAergic synapse | 0.856 |
| Organismal Systems;Nervous system;Glutamatergic synapse | 0.914 |
| Organismal Systems;Nervous system;Retrograde endocannabinoid signaling | 0.468 |
| Organismal Systems;Nervous system;Serotonergic synapse | 0.835 |

Table S12 Comparison of the inflammation index of participants between GDM group and ND group

|  | GDM group (n=11) | ND group (n=82) | *t or χ^2^* | *P* |
| --- | --- | --- | --- | --- |
| Premature rupture of membrane, n (%) | 2 (18.18) | 14 (17.07) | - | 1.000*^*^* |
| Preterm delivery, n (%) | 1 (9.09) | 2 (2.44) | - | 0.318*^*^* |
| Intrauterine infection , n (%) | 0 (0.00) | 1(1.22) | - | 1.000*^*^* |
| White blood cell level in the first trimester (× 10^9^/L) | 8.79±2.46 | 8.55±1.87 | 0.378 | 0.706 |
| White blood cell level in the second trimester (× 10^9^/L) | 9.94±2.31 | 9.62±2.34 | 0.426 | 0.671 |
| Neutrophil percentage in the first trimester (%) | 69.22±6.48 | 70.19±6.37 | 0.475 | 0.636 |
| Neutrophil percentage in the second trimester (%) | 71.28±6.17 | 72.17±5.84 | 0.470 | 0.639 |

GDM: gestational diabetes mellitus; ND: non-diabetics; ^*^ by Fisher’s exact test.


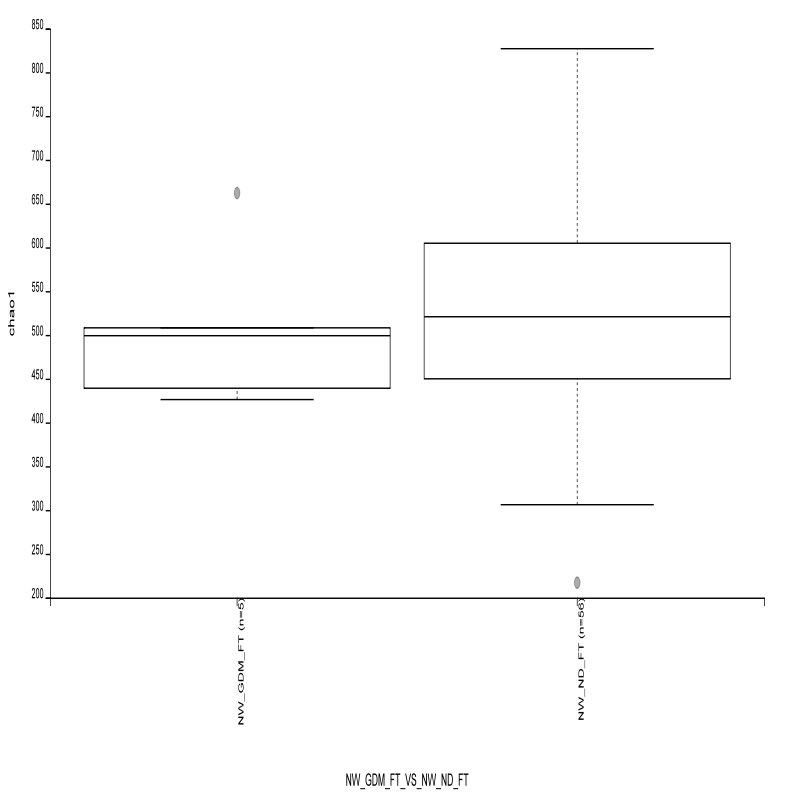
Figure S1 Comparison of the chao1 index of the gut microbiota between the GDM and ND group in the participants with normal weight in the first trimester

P=0.693


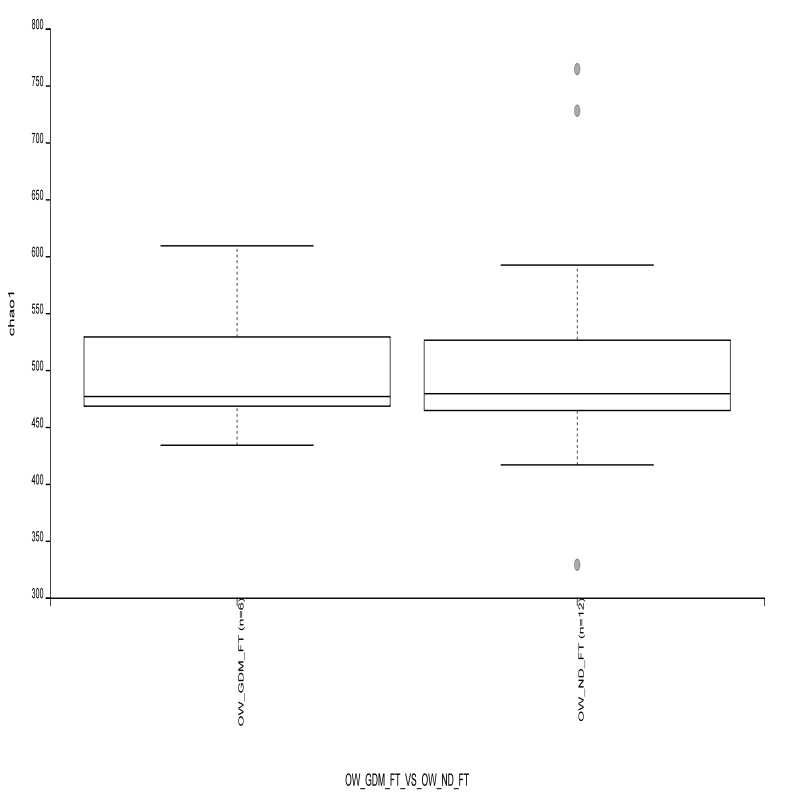
Figure S2 Comparison of the chao1 index of the gut microbiota between the GDM and ND group in the participants with overweight or obese in the first trimester

P=0.925


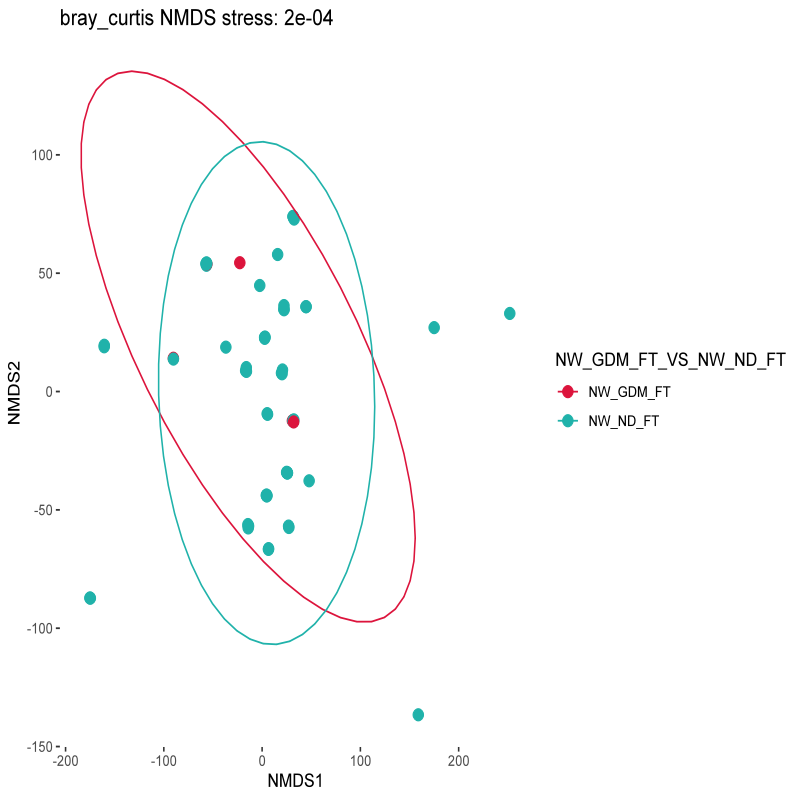


Figure S3 Comparison of the beta diversity of the gut microbiota between the GDM and ND group in the participants with normal weight in the first trimester


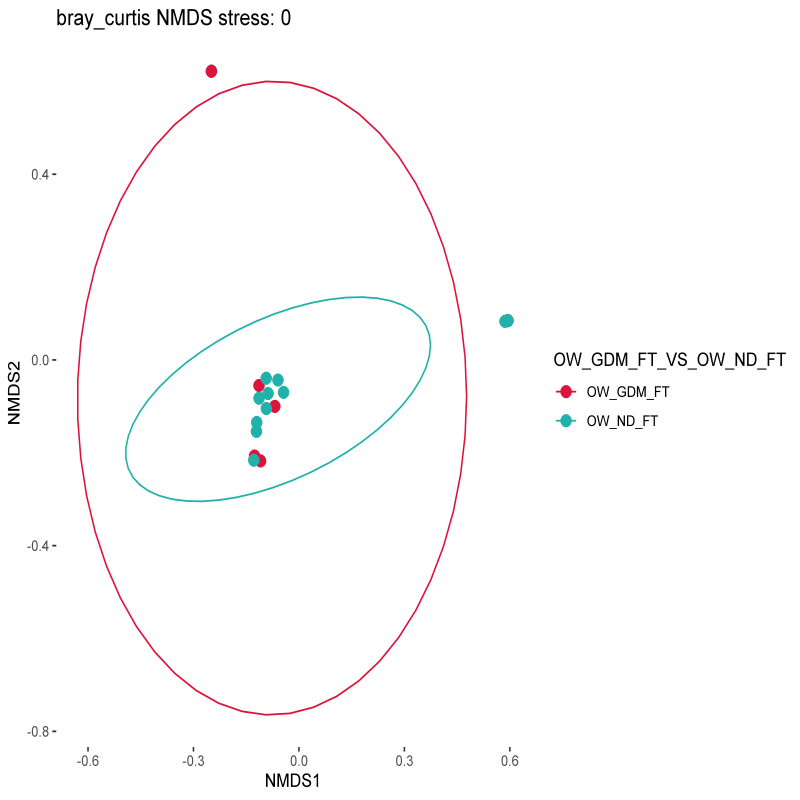


Figure S4 Comparison of the beta diversity of the gut microbiota between the GDM and ND group in the participants with overweight or obese in the first trimester


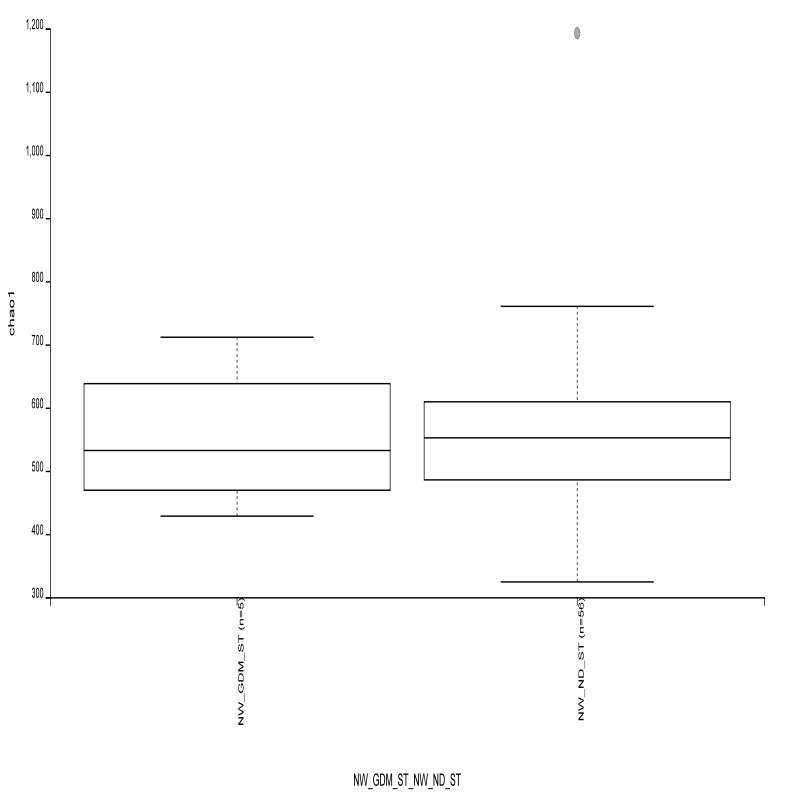
Figure S5 Comparison of the chao1 index of the gut microbiota between the GDM and ND group in the participants with normal weight in the second trimester

P=0.793


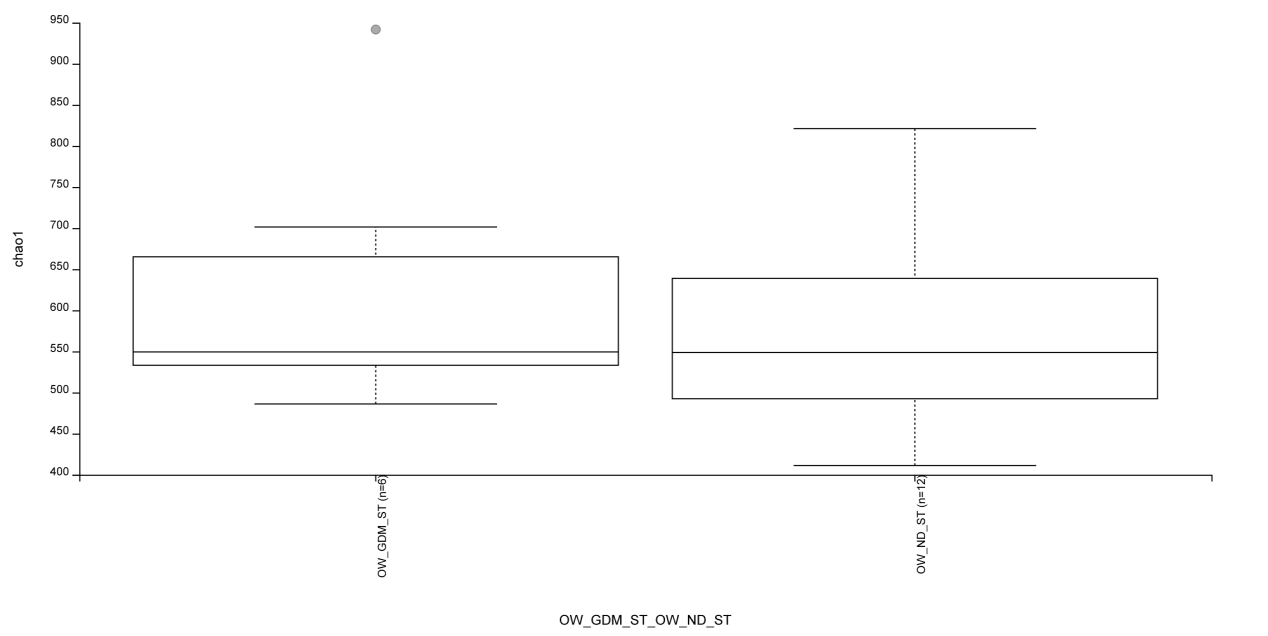
Figure S6 Comparison of the chao1 index of the gut microbiota between the GDM and ND group in the participants with overweight or obese in the second trimester

P=0.574


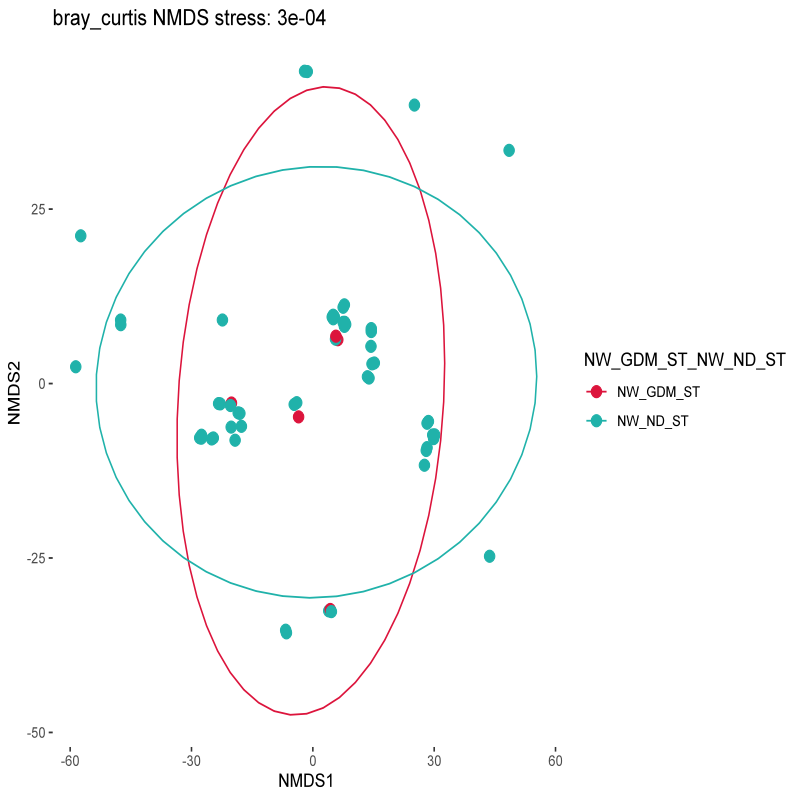


Figure S7 Comparison of the beta diversity of the gut microbiota between the GDM and ND group in the participants with normal weight in the second trimester


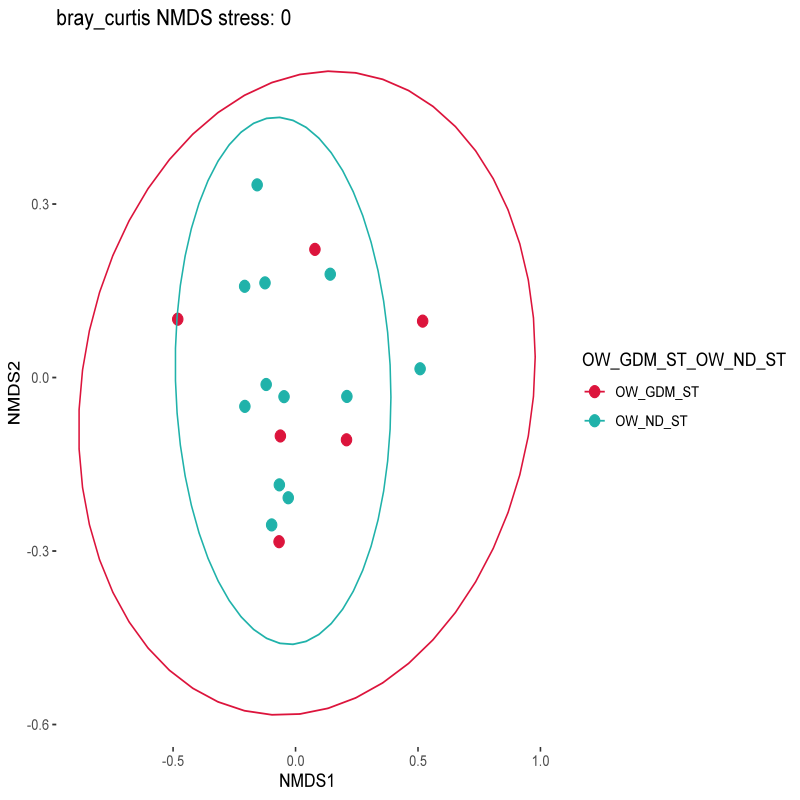
Figure S8 Comparison of the beta diversity of the gut microbiota between the GDM and ND group in the participants with overweight or obese in the second trimester


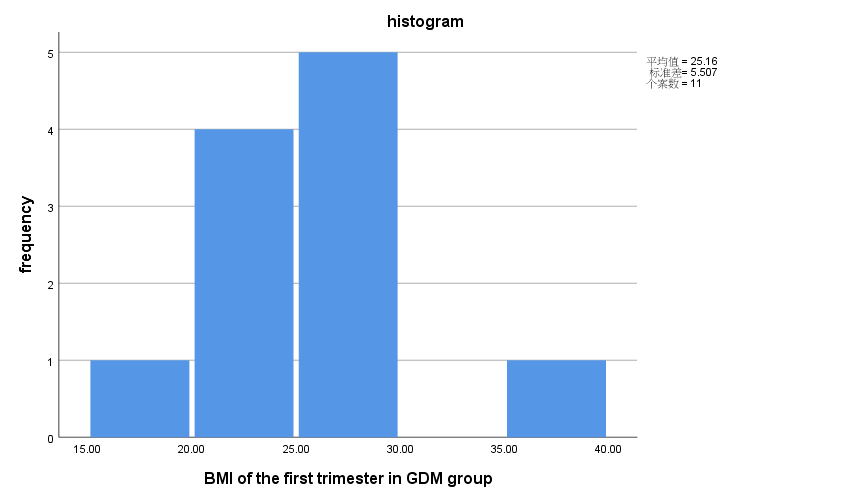


Figure S9 The histogram of the BMI of the first trimester in the GDM group


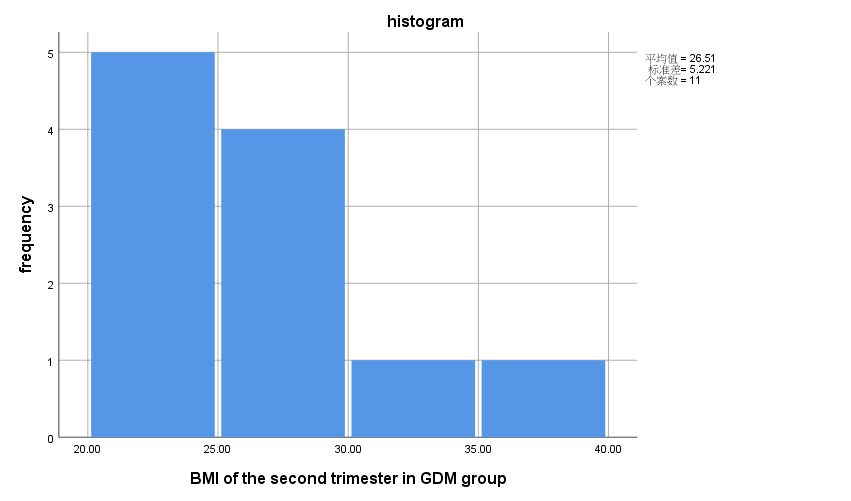


Figure S10 The histogram of the BMI of the second trimester in the GDM group


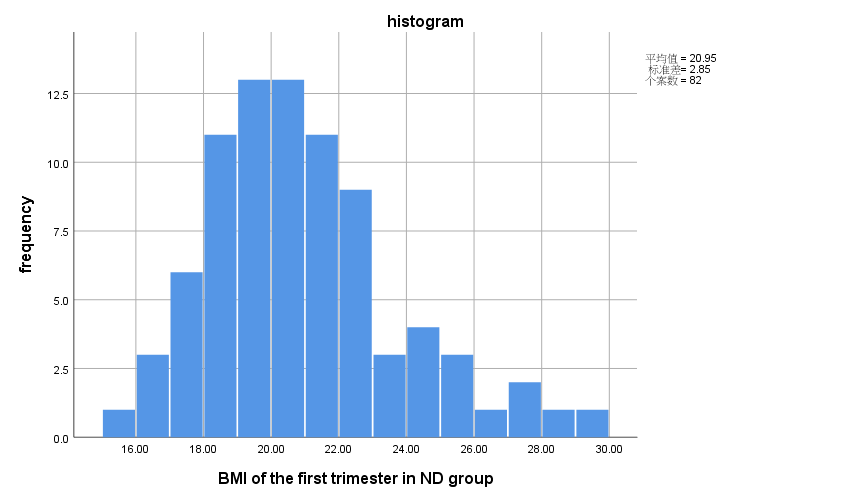


Figure S11 The histogram of the BMI of the first trimester in the ND group


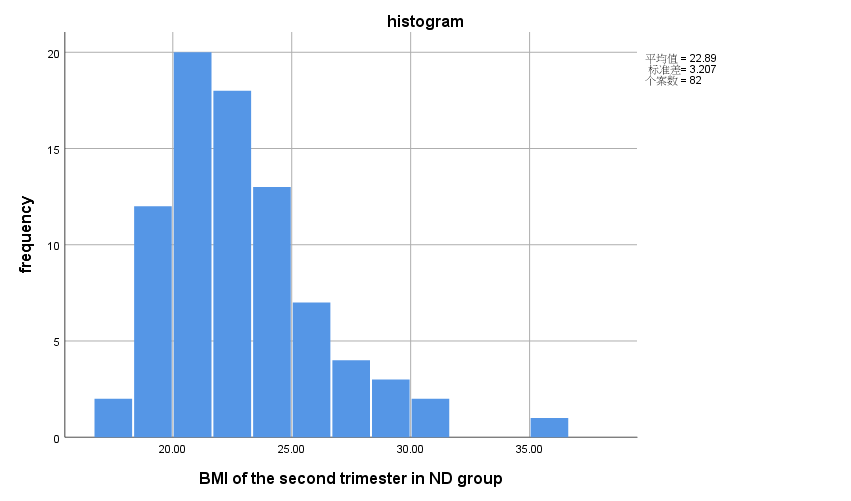


Figure S12 The histogram of the BMI of the second trimester in the ND group

**Document 1: Methodology of DNA extraction**

The operational steps for DNA extraction: Firstly, add isopropanol to the buffer GFA; add absolute ethanol to the deproteinization liquid RD and the washing liquid PWD. The volume to be added should refer to the label instructions on the bottle. Then, follow the steps below to extract the fecal flora DNA.

1. Sample processing Add 0.25 - 0.5 g of the sample into a 2 ml centrifuge tube. If it is a liquid sample, transfer 200 µl to the centrifuge tube. Add 500 µl of buffer SA, 100 µl of buffer SC and 0.25 g of grinding beads. (There may be residual RNA in fecal samples. If it is necessary to remove RNA, it is recommended to add 10 µl of RNase A (TIANGEN, RT405-02, provided by oneself)) Vortex and mix well or homogenize with a tissue homogenizer. Then heat and lyse at 70℃ for 15 minutes, followed by centrifugation at 12,000 rpm (~13,400×g) for 1 minute. Transfer the supernatant (approximately 500 µl) to a new 2 ml centrifuge tube. Note: For Gram-positive bacteria that are difficult to break the cell wall, increase the temperature to 95℃ to facilitate lysis.
2. Add 200 µl of buffer SH and mix well by vortexing for 5 seconds, and incubate at 4℃ for 10 minutes.
3. Centrifuge at 12,000 rpm (~13,400×g) for 3 minutes, transfer the supernatant to a new 2 ml centrifuge tube, add 500 µl of buffer GFA, and mix by inversion.
4. Add 10 µl of magnetic bead suspension G, and mix well by shaking for 5 minutes.
5. Place the centrifuge tube on a magnetic rack and let it stand for 30 seconds. After the magnetic beads are completely adsorbed, carefully aspirate the liquid.
6. Remove the centrifuge tube from the magnetic rack, add 700 µl of deproteinization liquid RD (please check whether absolute ethanol has been added before use), and mix well by shaking for 5 minutes.
7. Place the centrifuge tube on a magnetic rack and let it stand for 30 seconds. After the magnetic beads are completely adsorbed, carefully aspirate the liquid.
8. Remove the centrifuge tube from the magnetic rack, add 700 µl of washing liquid PWD (please check whether absolute ethanol has been added before use), and mix well by shaking for 3 minutes.
9. Place the centrifuge tube on a magnetic rack and let it stand for 30 seconds. After the magnetic beads are completely adsorbed, carefully aspirate the liquid.
10. Repeat steps 8 and 9 once each.
11. Place the centrifuge tube on the magnetic rack and air-dry at room temperature for 5 - 10 minutes.
12. Remove the centrifuge tube from the magnetic rack, add 50 - 100 µl of elution buffer TB, mix well by shaking, and incubate at 56°C for 5 minutes. During this period, shake and mix 3 times, 3 - 5 times each.
13. Place the centrifuge tube on a magnetic rack and let it stand for 2 minutes. After the magnetic beads are completely adsorbed, carefully transfer the DNA solution to a new centrifuge tube and store it under appropriate conditions.

**Document 2: Methodology of sequencing**

1. Flow cell preparation and bridge amplification:

The Illumina sequencing platform uses flow cells as the basis for sequencing. The flow cell surface is fixed with sequences complementary to the library adaptor, which enables the library to be attached to the flow cell surface. By bridge amplification, millions of clusters were formed on the surface of the flow cell for each library fragment. Each cluster is composed of the same DNA fragment.

2. Sequencing reaction: The sequencing reaction was carried out through cycles, and each cycle was added with a specific deoxyribonucleotide (dNTP) labeled with a different color fluorescent dye. Each dNTP also contains a reversible terminator that prevents sequential nucleotide addition.

3. Image capture and data analysis:

After each cycle, the fluorescence signal of each cluster was recorded by capturing images of the flow cell using a high-resolution camera. Nucleotides added to each cluster were determined from the fluorescence signal. Then, the reversible terminator is removed and ready for the next cycle.

4. Paired end sequencing:

For PE250 sequencing, one end of each cluster was first sequenced to generate a 250 base pair long read. The sequenced ends are then released from the flow cell by chemical or enzymatic treatment and the DNA strand is flipped so that the other end is facing up. The sequencing process was repeated for the other end to generate another 250 base pair long read.

**Document 3: Methodology of quality control**

In this study, the laboratory used the DADA2 plugin of Qiime2 to perform quality control on all the original sequences (input) of all samples, including filtering, denoising (correcting sequenced error sequences), merging, and removing chimeras to form ASVs. The quality control reference is set according to the following standards:

(1) Retained length of forward sequence: 249 (bases exceeding the 3' end will be truncated (low-quality bases), and 0 means no truncation).

(2) Retained length of reverse sequence: 249 (excess bases at the 3' end will be truncated (low-quality bases); this parameter is ignored in single-end mode).

(3) Forward primer: GTGCCAGCMGCCGCGGTAA (used to cut off the primer and previous base sequences in the forward reads).

(4) Reverse primer: CCGTCAATTCCTTTGAGTTT (used to cut off the primer and previous base sequences in the reverse reads. If not known, it can also be left blank; this parameter is ignored in single-end mode).

(5) Splicing overlap threshold: 12 (the forward and reverse sequences can be spliced only when the overlap region is greater than this threshold).

(6) Number of bases to be cut at the 5' end of the forward sequence: 25 (used to cut off low-quality bases at the 5' end of the forward sequence).

(7) Number of bases to be cut at the 5' end of the reverse sequence: 26 (used to cut off low-quality bases at the 5' end of the reverse sequence).

(8) Trimming quality value: 2 (check reads from the 5' end to the 3' end, cut off the first base less than or equal to this quality and the subsequent sequence. If the remaining sequence length is lower than the 'retained sequence length', then discard this sequence).

(9) Sampling denoising method: independent (denoising is performed independently for each sample).

(10) Number of reads used for training the error model: 1,000,000 (the larger the number, the more reliable the model, but the running time is also longer).
